# Replacing device‐measured sedentary time with physical activity is associated with lower risk of coronary heart disease regardless of genetic risk

**DOI:** 10.1111/joim.13715

**Published:** 2023-08-24

**Authors:** Youngwon Kim, Haeyoon Jang, Mengyao Wang, Qiaoxin Shi, Tessa Strain, Stephen J Sharp, Shiu Lun Au Yeung, Shan Luo, Simon Griffin, Nicholas J. Wareham, Katrien Wijndaele, Soren Brage

**Affiliations:** ^1^ School of Public Health The University of Hong Kong Li Ka Shing Faculty of Medicine Pokfulam Hong Kong; ^2^ MRC Epidemiology Unit University of Cambridge School of Clinical Medicine Cambridge United Kingdom

**Keywords:** coronary heart disease, genetic risk, physical activity, sedentary behavior, UK Biobank

## Abstract

**Background:**

Excess sedentary time (ST) is recognized as an important modifiable risk factor for coronary heart disease (CHD). However, whether the associations of genetic susceptibility with CHD incidence can be modified by replacing wearable‐device‐measured ST with physical activity (PA) is unknown.

**Objectives:**

To examine the associations of wearable‐device‐measured ST replaced by PA with incident CHD across strata of genetic susceptibility.

**Methods:**

This study included 77,500 White British (57% female) with valid wrist‐worn accelerometry and without prevalent CHD/stroke from UK Biobank. Genetic susceptibility to CHD was quantified through weighted polygenic risk scores for CHD based on 300 single‐nucleotide polymorphisms. Wrist‐worn accelerometer data were used to derive ST, light PA, and moderate‐to‐vigorous PA (MVPA).

**Results:**

Reallocation of 60 min/day of ST into the same amount of MVPA was associated with approximately 9% lower relative risk of CHD for all participants and across strata of genetic risk: replacement of 1 min/day of ST associated with <1% lower relative risk of CHD. No evidence of interaction (*p*: 0.784) was found between genetic risk and ST for CHD risk. Reallocating 60 min/day of ST into the same MVPA time was associated with greater absolute CHD risk reductions at high genetic risk (0.27%) versus low genetic risk (0.15%).

**Conclusions:**

Replacing any amount of ST with an equal amount of MVPA time is associated with a lower relative risk of CHD, irrespective of genetic susceptibility to CHD. Reductions in CHD absolute risk for replacing ST with MVPA are greater at high genetic risk versus low genetic risk.

## Graphical abstract

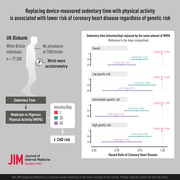

**Abbreviations**
CHD: coronary heart disease
ST: sedentary time
Light PA: light physical activity
MVPA: moderate‐to‐vigorous physical activity
PRS: polygenic risk scores
GWAS: genome‐wide association studies
SNPs: single‐nucleotide polymorphisms
ENMO: Euclidean Norm Minus One
ILRs: isometric log‐ratio pivot coordinates
HR: hazard ratio

## Introduction

Preventing the onset of coronary heart disease (CHD) is a clinical priority [1]. Development of CHD is attributable to both genetic and nongenetic lifestyle behaviors. The underlying genetic etiology of CHD has been characterized through genome‐wide association studies (GWAS), which have identified an expansive list of single‐nucleotide polymorphisms (SNPs) associated with risk of CHD [2, 3]. This methodological advance allows the estimation of an individual's unique genetic susceptibility to CHD through the calculation of polygenic risk scores (PRS) based upon the known SNPs [4, 5], thereby facilitating the possibility of personalized approaches to CHD prevention [6, 7].

Of nongenetic, lifestyle behaviors, reducing time spent sedentary and replacing it with more active time have been recognized as a major behavioral target for the prevention of cardiovascular events, including CHD [8]. Compelling evidence indicates that greater sedentary time (ST) is associated with an increased risk of cardiovascular disease [9, 10, 11, 12], and reallocation of ST into physical activity (PA) is known to be associated with a reduced risk of cardiovascular mortality [13, 14, 15] and cardiometabolic risk markers [16, 17, 18]. As such, current public health guidelines [19] specifically recommend replacing ST with PA time. However, to the best of our knowledge, no previous research has taken into account an individual's genetic susceptibility to CHD in understanding the associations of wearable‐device‐measured ST with incident CHD outcomes [20]. Little is also known about the prospective associations of ST in conjunction with other time‐use movement behaviors (moderate‐to‐vigorous PA [MVPA] [21, 22, 23, 24], light physical activity [light PA] [25, 26], and sleep [27]) with risk of cardiovascular disease including CHD in the context of genetics. Given these mutually exclusive movement behaviors (e.g., ST, light PA, MVPA, and sleep) occur over a confined, 24‐hour period, changes in time spent in one movement behavior (e.g., ST) necessarily entail changes in time spent in other behaviors. This codependent nature of ST relative to the other movement behaviors is, therefore, critical to consider when understanding the contribution of ST and replacement of it by PA toward the development of cardiovascular disease [28]. Currently, whether replacing ST with PA time benefits CHD prevention to a greater degree in individuals at high genetic risk of CHD compared with those at low genetic risk of CHD is unknown. Our study aims to determine the associations of wearable‐device‐measured ST and its replacement by light PA or MVPA with incident CHD, across varying levels of genetic susceptibility to CHD using compositional isotemporal substitution modeling [29, 30].

## Methods

### Study design and participants

This study utilizes data from UK Biobank, an ongoing prospective cohort of >500,000 UK adults aged 40–69 years at recruitment. The inclusion criteria were being registered within the National Health Service and living in a place <25 mi away from 1 of 22 assessment centers across the United Kingdom. Baseline measurements (2006–2010) collected genetic, biological, and behavioral information, including smoking, dietary intake, activity, and sleep. Invitations to take part in the accelerometry sub‐study of UK Biobank (2013–2015) were sent to 236,519 participants who had provided an email address. A subset of 103,711 individuals received a mail package, containing an accelerometer device (Axivity AX3) to measure triaxial acceleration, preconfigured to a sampling rate of 100 Hz and a dynamic range of ±8 g. Participants were instructed to start wearing the accelerometer device on the dominant wrist for full 24 hours over the subsequent 7 days. Participants were asked to send the accelerometer back to the coordinating center after the 7‐day monitoring period. Information about the UK Biobank and accelerometry sub‐study is provided elsewhere [31, 32]. The UK Biobank study was approved by the Northwest Multi‐Centre Research Ethics Committee. The present analysis included 77,500 individuals (Fig. S1) who met the following inclusion criteria: sufficient valid accelerometry data, self‐identifying as “White British” (European ancestry), not being removed from principal component analysis as an ethnic outlier, no prevalence of cardiovascular disease before the analytical baseline (i.e., start of the accelerometry sub‐study) (Supplemental Text 1 and Fig. S1), no missing covariates, and incident CHD cases accrued after the first year follow‐up.

### Exposures

#### Genetic susceptibility to CHD

In the UK Biobank, genotyping was performed on all participants using the UK Biobank Axiom Array and UK BiLEVE Axiom Array, with imputation to a reference panel of HRC in combination with UK10K [33]. We estimated genetic susceptibility to CHD in the form of weighted PRS for CHD [3]. Detailed information about the procedure is described elsewhere [3]. Briefly, the construction of PRS was based on 300 uncorrelated SNPs [2] for CHD risk (Table S1). The 300 SNPs are a combination of genome‐wide significant SNPs and uncorrelated SNPs at a false discovery rate of 5%, the latter of which were identified through a meta‐analysis of the CARDIoGRAMplusC4D 1000 Genomes‐imputed GWAS or MIGen/CARDIoGRAM Exome chip study and interim UK Biobank genotype data [3]. A weighted PRS for each participant was quantified by summing the number of risk alleles, multiplied by the established respective effect estimates [2, 3]. The calculated weighted PRS showed a normal distribution (Fig. S2) and was categorized into low, middle, and high genetic risk of CHD, according to the tertiles. The present study did not include any ambiguous palindromic variants.

#### Wearable‐device‐measured movement behaviors

Raw accelerometry data collected were calibrated to local gravity (1*g*) with temperature compensation [34] and filtered to dampen machine noise using a fourth‐order Butterworth low‐pass filter with a cut‐off (3 dB) frequency of 20 Hz. To isolate the movement‐related acceleration from gravitational acceleration, the Euclidean Norm Minus One (ENMO) metric was calculated as the Euclidean Norm of acceleration in three axes minus one gravitational unit, with negative values truncated to zero. Previous research has demonstrated a valid estimation of activity intensity [35] and energy expenditure [36, 37], and their association with mortality [38] through wrist accelerometry. Compared with waist accelerometry, wrist accelerometry is being increasingly adopted as a more common method for monitor wear and tends to better capture upper body movements (e.g., weight lifting), whereas parameter estimation methods have yet to be standardized [39]. Non‐wear was identified as time periods of ≥60 min where standard deviations of the three axes were all <13.0 mg (1 mg= 0.001 g) [40]. ENMO was classified into ST (<30 mg, excluding self‐reported sleep time, assessed through a question, “About how many hours sleep do you get in every 24 hours? (please include naps)”), light PA (30–125 mg) and MVPA (≥125 mg) [26, 38, 41, 42, 43]. The present analysis was restricted to participants with >72 hours of wear time (across the 7 days), mean ENMO <500 mg, or ENMO values recorded in each 1‐hour time block of the 24‐hour cycle [42, 44]. Three sets of isometric log‐ratio pivot coordinates (ILRs) (e.g., ST, light PA, and MVPA) were computed to quantify the contribution of one compositional behavior relative to other compositional behaviors (Supplemental Methods 1) [30, 45, 46]. Sleep was used for computing the coordinates but not included in the statistical models given its nonlinear, U‐shaped association with cardiovascular disease [47].

### Incidence of CHD

Sources of CHD incident data included the national death registry and hospital episode statistics. Codes of International Classification of Diseases (ICD) were used to adjudicate CHD cases (ICD‐9: 410–411, 412.X, ICD‐10: I21–I24, I25.2) accrued until December 9, 2022 for individuals in England and Wales and December 19, 2022 for individuals in Scotland. Incident CHD was defined as the first observation of CHD events that occurred over a median 8.1‐year follow‐up (interquartile range: 7.6–8.7 years), resulting in 1421 incident CHD cases.

### Confounders

We included the following confounders [48]: sex, smoking status (never, previous, current), alcohol consumption (never, previous, currently <3 times/week, currently ≥3 times/week), diet (a combined score based on the number of intake of vegetable, fruit, fish, and processed and red meat [inverted], with a greater score indicating a more favorable dietary habit), Townsend Deprivation Index (a composite score of deprivation based on four variables: unemployment, non‐car ownership, nonhome ownership, and household overcrowding, with a greater score indicating a greater degree of deprivation), use of antihypertensive medication, use of blood‐glucose lowering medication, use of cholesterol‐lowering medication, genotyping array type (UK Biobank Axiom Array, UK BiLEVE Axiom Array), and the first 10 principal components of ancestry (to control for population stratification) [49].

### Statistical analyses

Cox regression with age (at the start of the accelerometry sub‐study) as the underlying timescale (not as a confounder) using compositional isotemporal substitution modeling was used to (1) estimate the associations of ST relative to other movement behaviors with incident CHD and (2) explore how reallocation of time spent in ST into light PA or MVPA is associated with changes in hazard of CHD [29, 46], given the interpretation of raw parameter estimates for ILRs is not intuitive [30, 45]. Each ILR pivot coordinate (e.g., ST, light PA, and MVPA; except sleep) was included in the model [46]. Plots were generated using the parameter estimates from compositional isotemporal substitution to show changes in predicted estimates of CHD risk resulting from the reallocation of ILRs of ST into ILRS of MVPA or light PA. A ternary heat map plot was used to describe the simplex space, with the compositional center placed at the center of the plot. Total variance within the matrices was calculated by dividing the sum of the variances on either side of the diagonal by *D* = 4 [50, 51]. Cox regression models using PRS as a continuous variable and a 3‐level categorical variable were fit to estimate the association of genetic risk with incident CHD with adjustment for sex, genotyping array type, and the first 10 principal components; we used PRS not only as a continuous variable to ensure sufficient statistical power, but also as a 3‐level categorical variable to show CHD risk that may vary across levels of genetic risk of CHD. Cumulative hazards of CHD were estimated for each category of genetic risk at all ages. Multiplicative interaction among three compositional parts, including ST and PRS for CHD, was tested in the models adjusted for confounders. Models were stratified by genetic susceptibility to CHD. Estimates of 8‐year absolute risk of CHD were calculated for all participants and across the three genetic risk categories for no replacement of ST and replacement of ST by MVPA, separately; absolute risk reduction was calculated as a difference between estimates of 8‐year absolute risk for no replacement of ST and those for replacement of ST by MVPA. All models were fit adjusting for second‐degree genetic relatedness (defined as kinship coefficients of 0.0442–0.0884) [52] by estimating cluster‐robust standard errors [33]. Log–log plots showed that the proportional hazards assumption was met for each covariate. The following six sensitivity analyses were performed: (1) excluding an additional 1 year of follow‐up to address the potential for reverse causality, (2) excluding individuals with the second‐degree genetic relatedness, (3) using alternative movement intensity (ENMO) cutpoints to define movement behaviors (e.g., ≤25 mg for ST and ≥125 mg for MVPA; and ≤35 mg for ST and ≥125 mg for MVPA), (4) using missing values imputed using multiple imputation by chained equations, (5) using PRS calculated based on 46 lead SNPs after applying a more stringent LD cut‐off point [53] of 0.001 in order to minimize overestimation of effect size of SNPs [54, 55] (Table S1), and (6) excluding individuals with cancer at baseline. The estimation of the weighted PRS was performed using PLINK2.0. Statistical analyses were performed using R Studio and Stata/MP Version 16.0 (StataCorp LP).

## Results

Participants’ demographic information(*n* = 77,500; 57% female; mean age of 56.3 years) is presented in Table S2. The arithmetic geometric means of ST, light PA, and MVPA were 633.0, 287.2, and 64.2 min/day, respectively.

Figure S3 shows the distribution of individuals for relative proportions of time allocated across three movement behaviors (including ST, light PA, and MVPA) within an equilateral triangle. The vast majority (74.5%) of observations lay around the bottom right corner of the plot where the relative time was greater than 40% for ST, less than 50% for light PA, and less than 20% for MVPA.

**Fig. 1:**
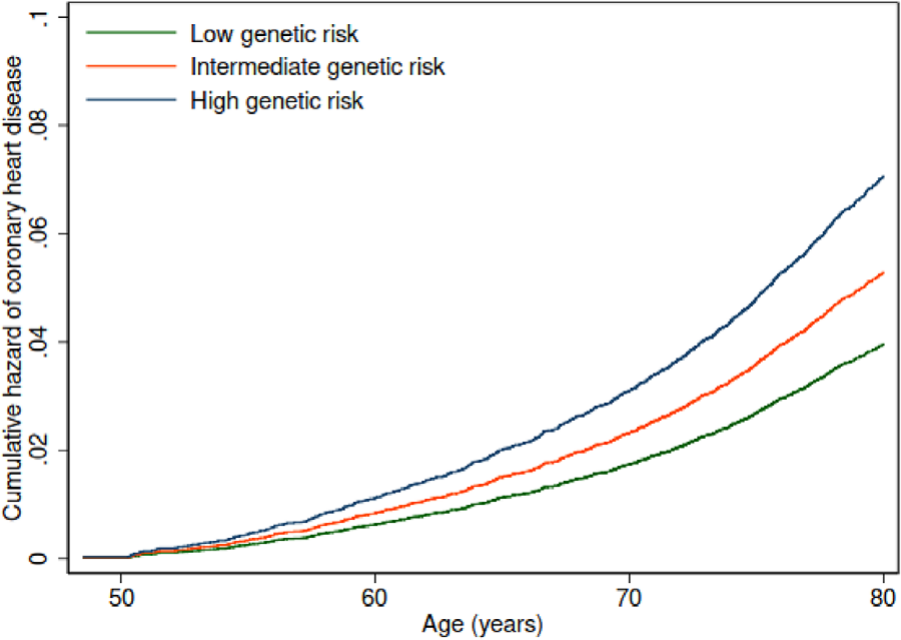
Cumulative hazards of coronary heart disease (CHD) for each genetic risk category by age. Notes: Cox regression models using age (at the start of the accelerometry sub‐study) as the underlying timescale (not as a confounder) were adjusted for sex, genotype array type, and first 10 principal components of genetic ancestry.

Figure 1 shows cumulative hazard functions of CHD for each category of genetic risk by age. Hazards of CHD were higher for high or intermediate genetic risk compared with low genetic risk at all ages. Table 1 shows the associations of genetic risk of CHD and ST (relative to other movement behaviors) with incident CHD. The hazard ratio (HR) of CHD was 1.30 (95% confidence interval; 1.02–1.68) for ST relative to other movement behaviors after adjusting for all confounders. An additional adjustment for PRS (Model 2) slightly attenuated the HR to 1.29 (1.01–1.66). The HR of CHD per one‐unit increment in PRS was 1.63 (1.49–1.78). The HRs of CHD were 1.34 (1.16–1.54) for intermediate genetic risk and 1.88 (1.65–2.15) for high genetic risk, compared with low genetic risk. There was no evidence of interaction between PRS and ST relative to other movement behaviors (*p*: 0.784). Greater time spent in MVPA relative to other movement behaviors was associated with a lower hazard ratio of CHD (HR: 0.87, 0.82–0.92) after adjusting for confounders and PRS (Table 1). There was an inconsistent association for light PA with adjustment for confounders and PRS (HR: 1.43; 1.11–1.83). There was no evidence of interaction between PRS and light PA (*p*: 0.316) or MVPA (*p*: 0.417).

**Table 1:** Associations of genetic risk of coronary heart disease (CHD), sedentary time, moderate‐to‐vigorous physical activity, and light physical activity with incidence of CHD.

	Hazard ratio of coronary heart disease (95% CI)	
	Model 1	Model 2
Polygenic risk scores (PRS) for CHD	1.63 (1.49, 1.78)	
Tertiles of PRS for CHD		
Low genetic risk	1.00 (reference)	
Intermediate genetic risk	1.34 (1.16, 1.54)	
High genetic risk	1.88 (1.65, 2.15)	
ST relative to all other behaviors	1.30 (1.02, 1.68)	1.29 (1.01, 1.66)
Light PA relative to all other behaviors	1.41 (1.10, 1.80)	1.43 (1.11, 1.83)
MVPA relative to all other behaviors	0.87 (0.83, 0.91)	0.87 (0.82, 0.92)

*Notes*: Model 1 using PRS for CHD as exposure treating it as either a continuous variable or a categorical variable: adjusted for sex, genotype array type, and first 10 principal components of genetic ancestry. Model 1 using ST, light PA, or MVPA as exposure: adjusted for sex, smoking status (never, previous, current), alcohol consumption (never, previous, currently <3 times/week, currently 3 times/week), diet (a combined score based on the number of increased intake of vegetable, fruit, and fish and decreased intake of processed and red meat, with a greater score indicating a more favorable dietary habit), Townsend Deprivation Index (a composite score of employment, car ownership, home ownership, and household overcrowding; based on postcode, with higher values indicating a higher degree of deprivation), use of antihypertensive medication, use of blood‐glucose lowering medication, and use of cholesterol‐lowering medication, with mutual adjustment of the three compositional variables. Model 2 using ST as exposure: adjusted for all confounders in Model 1 with an additional adjustment for polygenic risk scores (the genotype array type and first 10 principal components of genetic ancestry).

Abbreviations: CI, confidence interval; Light PA, light physical activity; MVPA, moderate‐to‐vigorous physical activity; PRS, polygenic risk scores; ST, sedentary time.

**Fig. 2:**
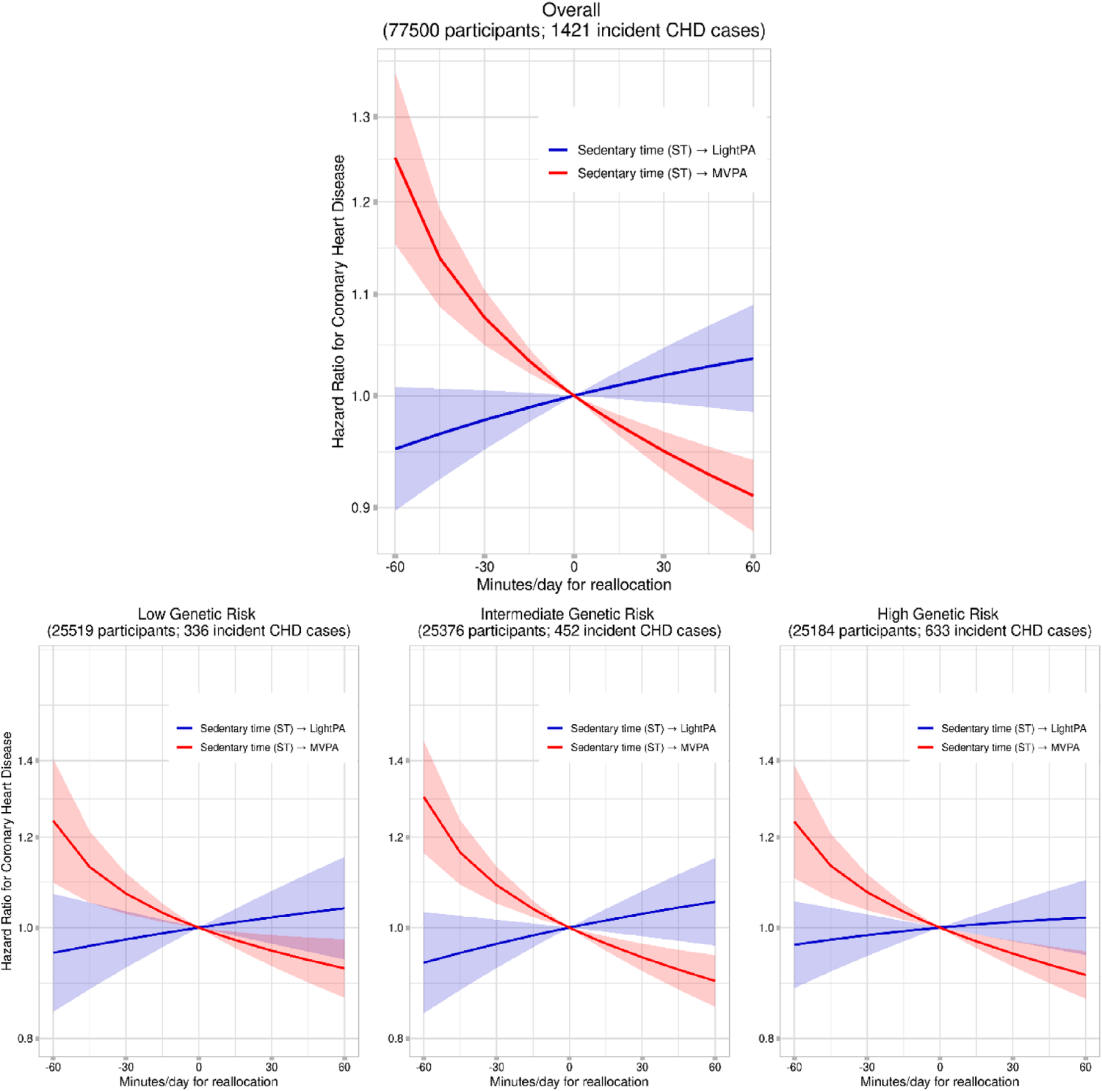
Reallocating sedentary time into physical activity for coronary heart disease. Notes: Cox regression models using age (at the start of the accelerometry sub‐study) as the underlying timescale (not as a confounder) were adjusted for sex, smoking status, alcohol consumption, diet, Townsend Deprivation Index, use of antihypertensive medication, use of blood‐glucose lowering medication, use of cholesterol‐lowering medication, genotype array type, and first 10 principal components of genetic ancestry, with mutual adjustment for the 3 ILR terms. Light PA, light physical activity; MVPA, moderate‐to‐vigorous physical activity; ST, sedentary time.

**Fig. 3:**
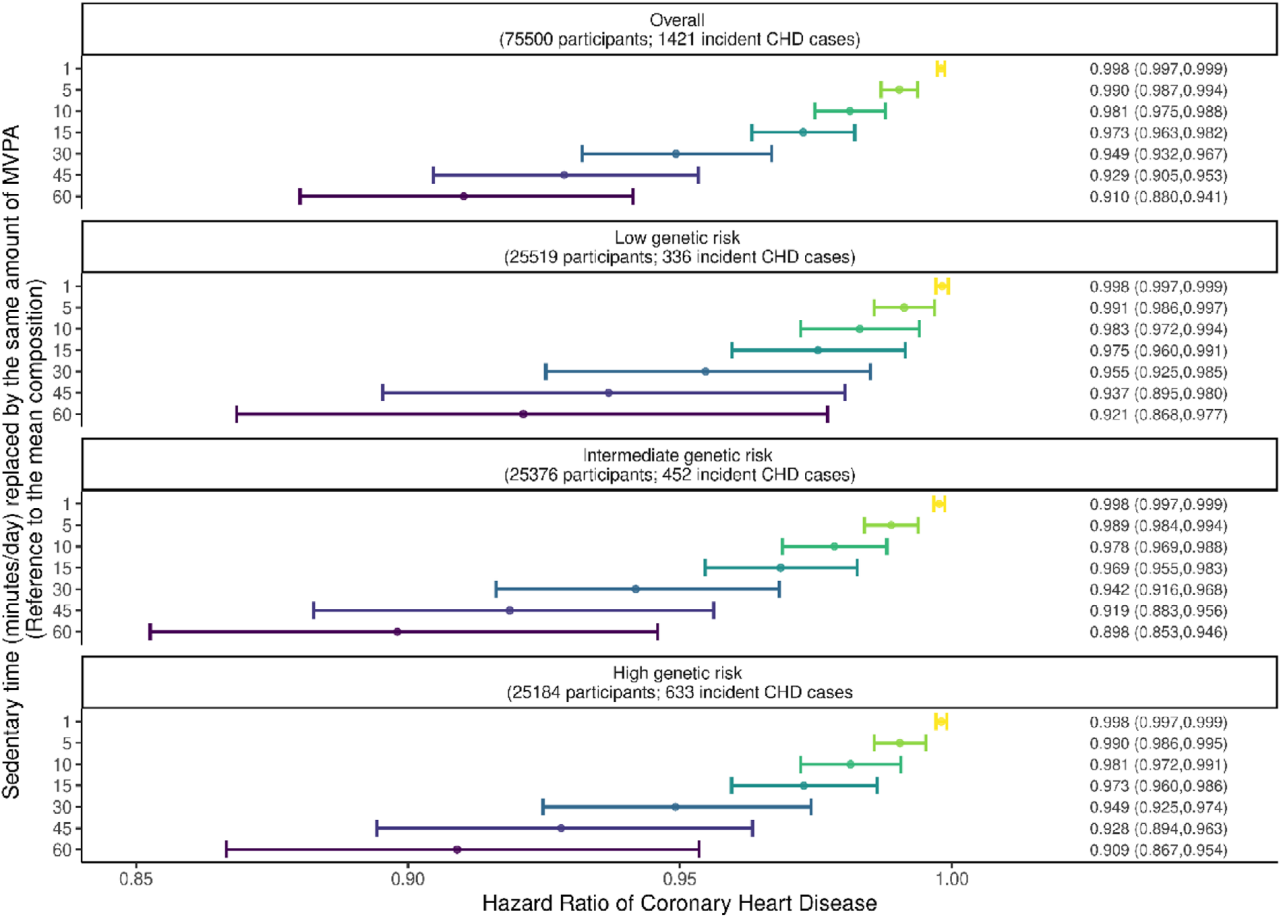
Potential impacts of replacing sedentary time with physical activity time. Notes: Cox regression models using age (at the start of the accelerometry sub‐study) as the underlying timescale (not as a confounder) were adjusted for sex, smoking status, alcohol consumption, diet, Townsend Deprivation Index, use of antihypertensive medication, use of blood‐glucose lowering medication, use of cholesterol‐lowering medication, genotype array type, and first 10 principal components of genetic ancestry, with mutual adjustment of the three 3 terms. CI, confidence interval; HR, hazard ratio; MVPA, moderate‐to‐vigorous physical activity; and ST, sedentary time.

Figure 2 shows the implications of reallocating ST into light PA or MVPA, and vice versa, on CHD hazards. Reallocating time spent sedentary into an equivalent amount of MVPA was associated with lower hazards of CHD overall, and across strata of genetic risk. However, there was no evidence that the replacement of ST with light PA time was associated with lower CHD hazards. Similarly, reallocating MVPA time (not light PA time) into an equal amount of ST was associated with increased CHD hazards across genetic risk strata. Similar patterns of associations were observed from the sensitivity analyses (Figs. S4–S9). Replacing any amount of ST with the corresponding amount of MVPA time was associated with lower hazards of CHD overall and across strata of genetic risk, including high genetic risk (Fig. 3). For example, although the CHD hazard was approximately 9% lower for reallocation of 60 min/day of ST into 60 min/day of MVPA, replacing as little as 1 min/day of ST was also associated with <1% lower CHD hazards.

**Fig. 4:**
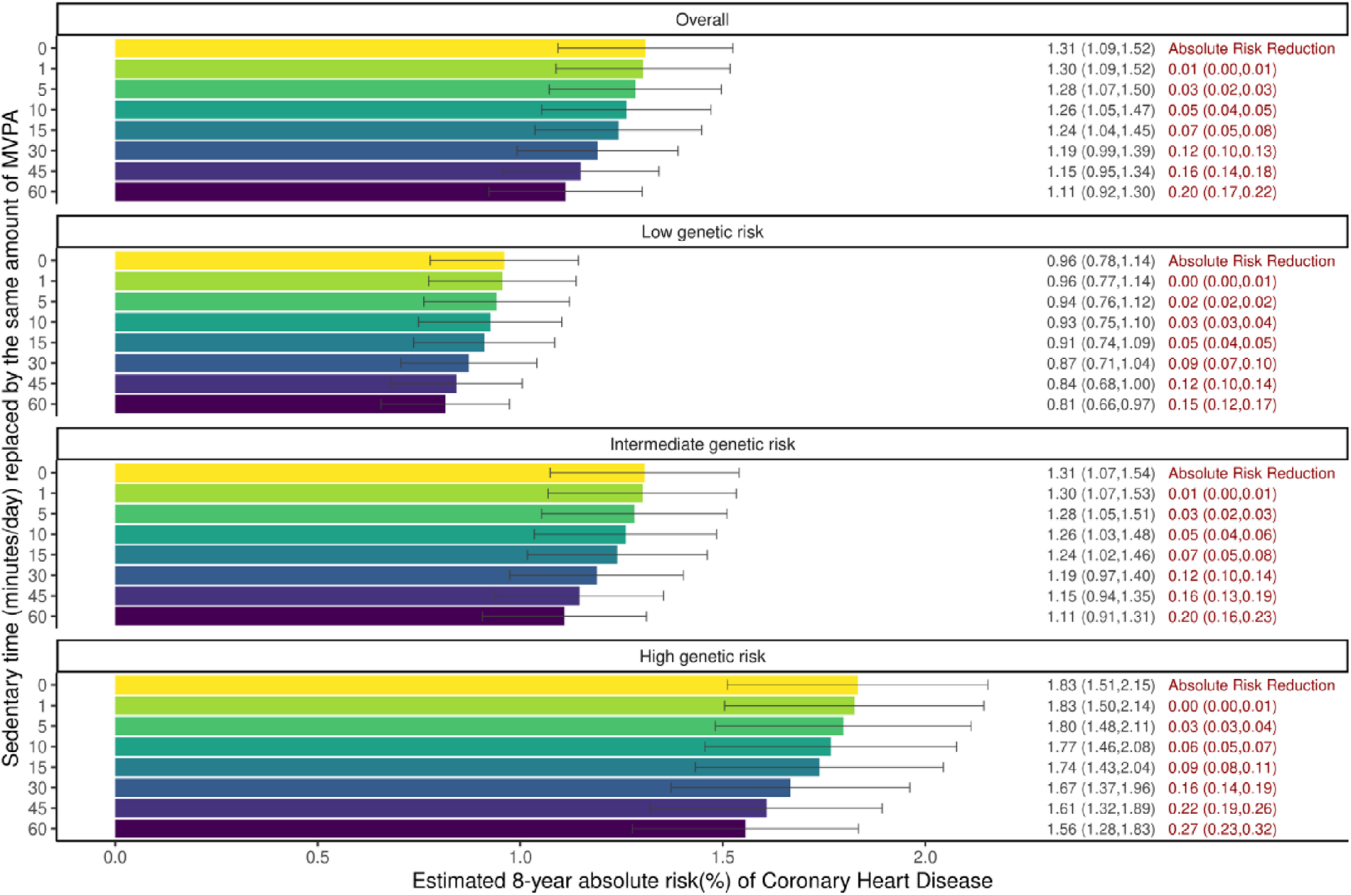
Estimated 8‐year absolute risk of coronary heart disease (CHD) and absolute risk reduction of CHD for sedentary time replacement. Notes: Cox regression models using age (at the start of the accelerometry sub‐study) as the underlying timescale (not as a confounder) were adjusted for sex, genotype array type, and the first 10 principal components of genetic ancestry. Two individuals who recorded less than 60 min/day of sedentary time are excluded in this analysis. CI, confidence interval; HR, hazard ratio; MVPA, moderate‐to‐vigorous physical activity; and ST, sedentary time.

Figure 4 shows estimated 8‐year absolute risk of CHD, and absolute risk reductions in CHD with no replacement of ST versus replacement of 1, 5, 10, 15, 30, and 60 min/day of ST by the same amount of MVPA overall and across genetic risk strata. For all participants, the 8‐year absolute risk of CHD was 1.31% which was reduced to 1.11% (an absolute risk reduction of 0.20%) by substituting 60 min/day of ST into 60 min/day of MVPA, with smaller absolute risk reductions in CHD through smaller amounts of ST replaced by MVPA. The absolute risk reductions in CHD resulting from the replacement of 60 min/day of ST by 60 min/day of MVPA were greater at high genetic risk (an absolute risk reduction of 0.27%; from 1.83% to 1.56%) compared with low (an absolute risk reduction of 0.15%; from 0.96% to 0.81%) or intermediate (an absolute risk reduction of 0.20%; from 1.31% to 1.11%) genetic risk.

## Discussion

This study is the first to investigate the potential role of replacing ST with physically active time in CHD prevention using integrated datasets of genotype and phenotype data including accelerometer‐derived activity profiles. Greater time spent sedentary relative to other movement behaviors was associated with increased risk of CHD across varying levels of genetic susceptibility to CHD. Importantly, reallocating any amount of ST into an equal amount of MVPA time was associated with lower CHD risk in individuals with high genetic susceptibility, as well as those with low genetic susceptibility.

Our study findings provide novel insights into the implications of reducing time spent sedentary in CHD prevention in individuals of different genetic susceptibilities. No previous research of ST as well as its replacement by active time took into consideration an individual's unique genetic predisposition and the codependent relationship of ST with other time‐use movement behaviors [13, 14, 15, 20]. The strong positive associations of ST with CHD risk (albeit effect estimates slightly modified by genetic susceptibility) imply that reduced time spent sedentary may benefit CHD prevention in individuals with high inherited CHD risk. There is compelling evidence that individuals with higher genetic susceptibility to CHD, as quantified through PRS [5], have a higher risk of developing CHD, as also shown in this study [3, 6, 7]. Hence, there will be a considerable clinical and public health benefit if individuals at high genetic risk can have a reduced risk of CHD simply by replacing ST with more active time [56].

Notably, our compositional isotemporal substitution modeling analyses demonstrated the potential benefits of any amount of ST replaced by the same amount of MVPA in CHD prevention across genetic risk strata. As large as a 9% lower CHD risk could be observed when substituting 60 min/day of ST with 60 min/day of MVPA across strata of genetic risk. However, reallocating as little as 1 min/day of ST into 1 min/day of MVPA was also associated with lower hazards of CHD. Of note, although the estimates of 8‐year CHD absolute risk were generally small, the replacement of ST by the same amount of MVPA was associated with a greater 8‐year CHD absolute risk reduction at high genetic risk (0.27% for 60‐min/day reallocation) compared with low (0.15% for 60‐min/day reallocation) or intermediate genetic risk (0.20% for 60‐min/day reallocation). This finding is similar to previous research that reported on larger reductions in absolute CHD risk corresponding to a per‐unit decrease in a lifestyle measure (e.g., lifestyle adherence and TV viewing) [7, 57]. This finding suggests that individuals at high genetic risk of CHD but replacing 60 min/day of ST with the same amount of MVPA may have greater reductions in absolute risk of developing CHD within the next 8 years compared with individuals at low genetic risk of CHD making the same 60‐min/day reallocation of ST into MVPA. Although the magnitude of 8‐year CHD absolute risk reductions observed herein may not be considered considerably large, the potential CHD absolute risk reductions through ST replaced by MVPA might likely be greater over an extended period of time (e.g., 20 years as opposed to 8 years), and, importantly, such implications might be more pronounced at high genetic risk than at low genetic risk. These findings are distinguishable from previous research that found favorable impacts of reallocating ST into MVPA time on intermediate cardiometabolic risk markers [16, 18, 58, 59], incident cardiovascular disease [60], or mortality [13, 61, 62] without taking into account genetic susceptibility. Taken together, our study informs future clinical trials of individuals with high genetic susceptibility to CHD aiming to reduce ST from a CHD‐prevention perspective.

The present study found no evidence of association for the replacement of ST by light PA for CHD incidence. This observation is consistent with the current evidence base that there is disease and mixed evidence on the association of light PA with cardiovascular mortality risk. For example, recent meta‐analysis and systematic review studies [25, 63] revealed that there was inconsistent evidence of benefits of reallocating ST into light PA or light PA alone relative to 14 out of 19 intermediate cardiometabolic risk markers [25], and in 18 out of 23 original studies included [63]. Another recent meta‐analysis of six prospective cohort datasets using compositional data analysis found ST and MVPA (but not light PA) to be associated with all‐cause mortality risk after mutual adjustment for each other [26]. Similar associations were observed in previous research using data from the National Health and Nutrition Examination Survey: no evidence of associations for light PA (albeit strong associations for ST and MVPA) in relation to mortality [64, 65]. A recent study using data from UK Biobank has also provided mixed findings that there was no evidence of association between incident CVD and light PA replaced proportionally by all other behaviors, but lower CVD incidence when replacing ST with light PA [60]. Nonetheless, no previous research [25, 26, 60, 63, 64, 65] has provided evidence on replacing ST with light PA for CHD incidence using compositional isotemporal substitution modeling across varying levels of genetic susceptibility, making it challenging to make a direct comparison with the present study. Further research is needed to determine the extent to which risk of CHD is prevented through replacement of ST by light PA as opposed to MVPA, and whether such associations vary by genetic susceptibility.

### Limitations

Several limitations of this research need to be considered. First, no causal inference can be made as this was an observational study. Moreover, this study is of European descendants who participated in the accelerometry sub‐study (Table S3), and UK Biobank participants have higher socioeconomic status and more favorable metabolic profiles, in general, than the average UK population [66]. Hence, findings may not be generalized to individuals of other ethnic groups or with subclinical symptoms. In addition, there may be reverse causation, but the sensitivity analysis, excluding the first 1 year of follow‐up, revealed similar findings as the main analysis. Another limitation is the inclusion of confounders assessed at baseline (between 2006 and 2010) in our analyses where the start of the accelerometry sub‐study (between 2013 and 2015) was used as the analytical baseline, a method used in previous research [38]. However, the confounders assessed at baseline remained generally stable (except medication use which increased with time) in a subset of 20,343 individuals who participated in both the baseline and first repeat‐assessment between 2012 and 2013, and in another subset of 56,014 individuals who participated in both the baseline and second repeat‐assessment in 2014 and thereafter (see Table S3). This observation is consistent with the observation of previous research [38]. Furthermore, unmeasured confounders or measurement error in the self‐reported confounders may mean that there is some residual confounding.

## Conclusion

Irrespective of genetic susceptibility to CHD, more time spent sedentary was associated with a higher risk of developing CHD. However, replacing any amount of ST (as little as 1 min/day) with the same amount of MVPA time was associated with lower risk of developing CHD across strata of genetic risk of CHD. Reductions in absolute risk of CHD from replacement of ST with MVPA were relatively greater at high versus low or intermediate genetic risk. Replacing time‐spent sedentary with MVPA should be a key behavioral target for the prevention of CHD in all individuals, particularly among those with high genetic susceptibility to CHD.

## Author contributions

Conceptualization; data curation; funding acquisition; investigation; methodology; project administration; resources; software; supervision; visualization; writing—original draft; writing—review and editing: Youngwon Kim. Conceptualization; data curation; formal analysis; investigation; methodology; software; validation; visualization; writing—review and editing: Haeyoon Jang. Data curation; formal analysis; investigation; project administration; resources; visualization; writing—review and editing: Mengyao Wang. Investigation; project administration; resources; visualization; writing—review and editing: Qiaoxin Shi. Investigation; validation; writing—review and editing: Tessa Strain. Formal analysis; investigation; methodology; visualization; writing—review and editing: Stephen J. Sharp. Data curation; investigation; methodology; resources; writing—review and editing: Shan Luo. Funding acquisition; investigation; writing—review and editing: Simon Griffin. Conceptualization; methodology; writing—review and editing: Katrien Wijndaele.

## Conflict of interest statement

All authors declare that there are no other conflicts of interest. All authors can take responsibility for the integrity of the data and the accuracy of the data analysis.

## Funding information

YK was supported by the U.S. National Academy of Medicine (NAM), Health and Medical Research Fund (HMRF) Research Fellowship [grant number 06200087], and Seed Grants of The University of Hong Kong Li Ka Shing Faculty of Medicine. NW, TS, KW, SJS, and SB are supported by UK Medical Research Council [grant numbers MC_UU_00006/1, MC_UU_00006/4, and MC_UU_12015/3]. NW and SB are supported by the NIHR Biomedical Research Centre in Cambridge (IS‐BRC‐1215‐20014). The NIHR Cambridge Biomedical Research Centre (BRC) is a partnership between Cambridge University Hospitals NHS Foundation Trust and the University of Cambridge, funded by the National Institute for Health Research (NIHR). The views expressed are those of the author(s) and not necessarily those of the NHS, the NIHR, or the Department of Health and Social Care. The funding body played no role in the collection, analysis, and interpretation of data; in the writing of the report; and in the decision to submit the article for publication.

## Supporting information


**Supplementary Table 1**. A list of 300 Single‐Nucleotide Polymorphisms (SNPs) known to be associatedwith coronary heart disease risk.
**Supplementary Table 2**. Characteristics of participants
**Supplementary Table 3**. Characteristics of participants in the original UK Biobank sample and different sub‐samples.
**Supplementary Figure 1**. Participant flow chart.
**Supplementary Figure 2**. Distribution of individuals by calculated weighted polygenic risk scores for coronary heart disease (CHD).
**Supplementary Figure 3**. A compositional ternary heat map plot indicating the distribution of individuals according to relative proportions of time spent on sedentary time, light physical activity, and moderate‐to‐vigorous physical activity.
**Supplementary Figure 4**. Hazard ratios of coronary heart disease estimated from reallocating sedentary time into physical activity time (and vice versa), while keeping the remaining components constant, after excluding an additional 1 year of follow‐up to address potential for reverse causality.
**Supplementary Figure 5**. Hazard ratios of coronary heart disease estimated from reallocating sedentary time into physical activity time and physical activity time into sedentary time, while keeping the remaining components constant after excluding individuals with the 2nd‐degree genetic relatedness.
**Supplementary Figure 6**. Hazard ratios of coronary heart disease estimated from reallocating sedentary time into physical activity time and physical activity time into sedentary time, while keeping the remaining components constant, using a different set of ENMO cut#x02010;offs to define the movement behaviors (Left panel: milli#x02010;g ≤25 for ST and milli#x02010;g ≥125 for MVPA; Right panel: milli#x02010;g ≤35 for ST and milli#x02010;g ≥125 for MVPA).
**Supplementary Figure 7**. Hazard ratios of coronary heart disease estimated from reallocating sedentary time into physical activity time and physical activity time into sedentary time, while keeping the remaining components constant, using missing values imputed using multiple imputation by chained equations.
**Supplementary Figure 8**. Hazard ratios of coronary heart disease estimated from reallocating sedentary time into physical activity time and physical activity time into sedentary time, while keeping the remaining components constant, using a weighted polygenic risk score calculated based only on 46 lead SNPs from 46 loci after applying a more stringent LD cut#x02010;off point of 0.001 in order to minimize over#x02010;estimation of effect size of SNPs.
**Supplementary Figure 9**. Hazard ratios of coronary heart disease estimated from reallocating sedentary time into physical activity time and physical activity time into sedentary time, while keeping the remaining components constant after excluding individuals with prevalent cancer at baseline
