# Supplementary material for: Replacing device‐measured sedentary time with physical activity is associated with lower risk of coronary heart disease regardless of genetic risk
Source: J Intern Med. 2023 Aug 24;295(1):38–50. doi: 10.1111/joim.13715 (PMC10953003; doi:10.1111/joim.13715)
Supplement: Supplementary file 1 — Supplementary Table 1. A list of 300 Single‐Nucleotide Polymorphisms (SNPs) known to be associatedwith coronary heart disease risk. Supplementary Table 2. Characteristics of participants Supplementary Table 3. Characteristics of participants in the original UK Biobank sample and different sub‐samples. Supplementary Figure 1. Participant flow chart. Supplementary Figure 2. Distribution of individuals by calculated weighted polygenic risk scores for coronary heart disease (CHD). Supplementary Figure 3. A compositional ternary heat map plot indicating the distribution of individuals according to relative proportions of time spent on sedentary time, light physical activity, and moderate‐to‐vigorous physical activity. Supplementary Figure 4. Hazard ratios of coronary heart disease estimated from reallocating sedentary time into physical activity time (and vice versa), while keeping the remaining components constant, after excluding an additional 1 year of follow‐up to address potential for reverse causality. Supplementary Figure 5. Hazard ratios of coronary heart disease estimated from reallocating sedentary time into physical activity time and physical activity time into sedentary time, while keeping the remaining components constant after excluding individuals with the 2nd‐degree genetic relatedness. Supplementary Figure 6. Hazard ratios of coronary heart disease estimated from reallocating sedentary time into physical activity time and physical activity time into sedentary time, while keeping the remaining components constant, using a different set of ENMO cut#x02010;offs to define the movement behaviors (Left panel: milli#x02010;g ≤25 for ST and milli#x02010;g ≥125 for MVPA; Right panel: milli#x02010;g ≤35 for ST and milli#x02010;g ≥125 for MVPA). Supplementary Figure 7. Hazard ratios of coronary heart disease estimated from reallocating sedentary time into physical activity time and physical activity time into sedentary time, while keeping the remaining components c [file JOIM-295-38-s001.docx]

**Supplemental Material**

[Title Page 1](#_Toc26812)

[Supplemental Text 1 2](#_Toc4594)

[Supplemental Table 1 3](#_Toc5947)

[Supplemental Table 2 8](#_Toc4594)

[Supplemental Table 3 9](#_Toc5947)

[Supplemental Methods 1 1](#_Toc4594)1

[Supplemental Figure 1 13](#_Toc5947)

[Supplemental Figure 2 1](#_Toc5947)4

[Supplemental Figure 3 1](#_Toc5947)5

[Supplemental Figure 4 1](#_Toc5947)6

[Supplemental Figure 5 1](#_Toc5947)8

[Supplemental Figure 6 20](#_Toc5947)

[Supplemental Figure 7 2](#_Toc5947)2

[Supplemental Figure 8 2](#_Toc5947)4

[Supplemental Figure 9 2](#_Toc5947)6

Supplemental Text 1. Definitions for prevalence of ***coronary heart disease (CHD) and stroke, and prevalence of cancer.***

***Definition for prevalence of coronary heart disease (CHD) and stroke***

Prevalence of coronary heart disease (CHD) and stroke was defined as having any record of CHD or stroke (based on combined data sources of national death registry and hospital admission records) at baseline or before the start of the accelerometry sub-study (e.g., analytical baseline), or having self-reported physician-diagnosis of CHD or stroke^1^ at baseline. The Codes of International Classification of Diseases (ICD) were used to adjudicate CHD (ICD-9: 410-411,412.X, ICD-10: I21-I24, I25.2) and stroke (ICD-9: 430.X, 431.X, 434.X, 434.0, 434.1, 434.9, 436.X; ICD-10: I60, I61, I63, I64.X), separately.

***Definition for prevalence of cancer***

Prevalence of cancer was defined as having any record of cancer (based on combined data sources of national death registry and hospital admission records, using ICD-10: C0-C97, D01-D48) or having self-reported physician-diagnosis of cancer^1^ before or at baseline.

Note: ^1^Participants were asked “Has a doctor ever told you that you have had cancer” and there were four choices for this question: “Yes”, “No”, “Do not know “and “Prefer not to answer” (UK Biobank field IDs: 2453). Participants were considered to have self-reported cancer if their answer for this question was “Yes.”

Supplemental Table 1. A list of 300 Single-Nucleotide Polymorphisms (SNPs) known to be associated with coronary heart disease risk.

| **SNPs** | **Effect allele** | **Other allele** | **Beta** | **P value** |
| --- | --- | --- | --- | --- |
| rs2843152 | C | G | 0.042 | 1.34E-05 |
| rs35465346 | G | A | 0.055 | 5.91E-06 |
| rs7538207 | C | T | 0.098 | 5.73E-06 |
| rs11811081 | C | A | 0.077 | 5.65E-05 |
| rs12733730 | A | G | 0.046 | 1.79E-05 |
| rs11485595 | T | C | 0.04 | 4.00E-06 |
| rs34232196 | C | T | 0.055 | 2.87E-08 |
| rs11591147^#^ | G | T | 0.221 | 2.84E-10 |
| rs17111652 | T | C | 0.082 | 1.34E-05 |
| rs6665249 | A | G | 0.042 | 1.54E-05 |
| rs56170783^#^ | A | C | 0.104 | 2.14E-12 |
| rs2149821 | A | T | 0.039 | 3.17E-05 |
| rs10890013 | T | C | 0.035 | 2.26E-05 |
| rs113832197 | T | C | 0.081 | 3.40E-05 |
| rs7528419^#^ | A | G | 0.109 | 3.77E-27 |
| rs11552449 | T | C | 0.045 | 3.90E-05 |
| rs10305649 | A | C | 0.114 | 2.87E-06 |
| rs11810571 | G | C | 0.058 | 2.21E-08 |
| rs6689306^#^ | A | G | 0.05 | 1.46E-09 |
| rs2789422 | G | A | 0.035 | 4.63E-05 |
| rs6413828 | A | T | 0.038 | 1.77E-05 |
| rs183692864 | G | A | 0.244 | 5.49E-05 |
| rs1892094 | C | T | 0.02 | 1.55E-02 |
| rs6700559 | C | T | 0.022 | 6.96E-03 |
| rs2820315 | T | C | 0.043 | 2.09E-06 |
| rs67180937^#^ | G | T | 0.071 | 8.45E-14 |
| rs17464857 | T | G | 0.06 | 6.90E-07 |
| rs3755549 | C | T | 0.034 | 2.64E-05 |
| rs2709437 | T | C | 0.034 | 4.38E-05 |
| rs16986953^#^ | A | G | 0.105 | 4.77E-10 |
| rs585967^#^ | C | A | 0.066 | 2.76E-08 |
| rs58560619 | C | T | 0.036 | 1.65E-05 |
| rs4299376^#^ | G | T | 0.055 | 5.65E-10 |
| rs4076834 | T | G | 0.1 | 1.23E-08 |
| rs139591697 | T | C | 0.102 | 5.15E-05 |
| rs72375964 | A | G | 0.035 | 5.01E-05 |
| rs7568458^#^ | A | T | 0.061 | 2.39E-13 |
| rs149366039 | T | C | 0.731 | 4.03E-05 |
| rs7578433 | T | C | 0.07 | 3.87E-05 |
| rs79716828 | C | A | 0.111 | 8.63E-06 |
| rs6761276 | T | C | 0.036 | 2.66E-05 |
| rs7570006 | C | T | 0.048 | 3.78E-05 |
| rs17678683 | G | T | 0.077 | 1.15E-07 |
| rs35500812 | A | C | 0.042 | 3.20E-06 |
| rs12619842 | G | C | 0.048 | 8.57E-06 |
| rs62172372 | A | G | 0.046 | 3.61E-05 |
| rs114123510^#^ | A | T | 0.118 | 2.88E-19 |
| rs2011559 | G | A | 0.054 | 2.44E-05 |
| rs1250229^#^ | T | C | 0.069 | 1.85E-13 |
| rs2161967 | T | G | 0.039 | 6.21E-06 |
| rs2972146 | T | G | 0.047 | 6.50E-08 |
| rs10168194 | C | G | 0.039 | 6.78E-06 |
| rs13003675 | T | C | 0.042 | 1.72E-06 |
| rs10929113 | C | T | 0.042 | 5.24E-05 |
| rs143803699 | G | C | 0.12 | 2.95E-05 |
| rs748431 | G | T | 0.041 | 9.04E-07 |
| rs3821396 | G | A | 0.06 | 6.57E-06 |
| rs7623687^#^ | A | C | 0.072 | 3.72E-09 |
| rs77622129 | A | G | 0.094 | 2.00E-05 |
| rs62253653 | A | G | 0.038 | 4.35E-05 |
| rs71331765 | G | C | 0.051 | 4.47E-05 |
| rs6787409 | C | T | 0.039 | 1.63E-05 |
| rs9818870^#^ | T | C | 0.068 | 7.82E-09 |
| rs4632520 | C | T | 0.038 | 2.90E-05 |
| rs12493885^#^ | C | G | 0.071 | 3.29E-08 |
| rs10513507 | C | T | 0.036 | 2.20E-05 |
| rs34229028 | G | A | 0.037 | 1.25E-05 |
| rs9869263 | G | A | 0.048 | 3.74E-05 |
| rs113148244 | G | T | 0.144 | 2.20E-05 |
| rs16994919 | A | G | 0.06 | 9.55E-06 |
| rs2616407 | C | T | 0.049 | 5.60E-06 |
| rs13134452 | C | T | 0.037 | 3.16E-05 |
| rs72627509 | G | C | 0.054 | 8.10E-08 |
| rs10857147^#^ | T | A | 0.054 | 8.96E-09 |
| rs138495951 | G | A | 0.16 | 3.84E-05 |
| rs7678555 | C | A | 0.048 | 1.43E-07 |
| rs144059514 | G | A | 0.085 | 4.50E-03 |
| rs13109172 | C | T | 0.038 | 5.73E-06 |
| rs4593108 | C | G | 0.058 | 1.95E-08 |
| rs6841581^#^ | A | G | 0.068 | 4.57E-10 |
| rs7435973 | G | A | 0.059 | 4.51E-07 |
| rs3796587^#^ | C | G | 0.063 | 1.24E-09 |
| rs869396 | C | A | 0.039 | 1.85E-06 |
| rs11728590 | G | T | 0.037 | 1.97E-05 |
| rs71600236 | C | G | 0.039 | 6.54E-06 |
| rs112941079 | A | G | 0.059 | 3.86E-06 |
| rs5868014 | G | A | 0.052 | 1.04E-06 |
| rs111777100 | A | G | 0.093 | 5.35E-05 |
| rs288187 | C | T | 0.046 | 2.63E-05 |
| rs1800449 | T | C | 0.056 | 4.06E-07 |
| rs1500187 | G | A | 0.037 | 9.73E-06 |
| rs6883598 | C | A | 0.039 | 2.32E-05 |
| rs273909 | G | A | 0.053 | 9.94E-04 |
| rs251023 | G | A | 0.038 | 8.34E-06 |
| rs11955380 | C | A | 0.06 | 1.28E-05 |
| rs3776307 | G | A | 0.038 | 9.40E-06 |
| rs6860540 | G | A | 0.035 | 5.49E-05 |
| rs9501744 | C | T | 0.064 | 1.08E-06 |
| rs421329 | C | T | 0.049 | 1.55E-06 |
| rs742115 | C | T | 0.036 | 2.86E-05 |
| rs6458138 | G | A | 0.065 | 6.13E-05 |
| rs9349379^#^ | G | A | 0.105 | 9.95E-36 |
| rs13200993^#^ | T | C | 0.05 | 5.60E-09 |
| rs3130683 | T | C | 0.077 | 2.77E-08 |
| rs9268402 | A | G | 0.014 | 1.28E-01 |
| rs4472337 | T | C | 0.055 | 2.42E-06 |
| rs17609940 | G | C | 0.029 | 7.11E-03 |
| rs56015508 | C | A | 0.054 | 1.08E-07 |
| rs1214752 | C | T | 0.039 | 3.26E-06 |
| rs6905288 | A | G | 0.039 | 3.26E-06 |
| rs1330633 | G | A | 0.067 | 5.89E-05 |
| rs194937 | A | G | 0.048 | 1.26E-05 |
| rs11153071 | G | A | 0.05 | 3.00E-06 |
| rs9398803 | A | G | 0.034 | 6.23E-05 |
| rs12202017^#^ | A | G | 0.066 | 6.02E-14 |
| rs9493752 | A | G | 0.154 | 7.05E-06 |
| rs2492304 | A | T | 0.033 | 5.59E-05 |
| rs2153219 | A | G | 0.051 | 2.59E-06 |
| rs55730499^#^ | T | C | 0.268 | 5.64E-49 |
| rs186696265 | T | C | 0.466 | 8.97E-36 |
| rs4252198 | G | C | 0.152 | 1.66E-05 |
| rs79018195 | C | T | 0.304 | 1.26E-06 |
| rs41269133 | T | C | 0.079 | 4.78E-08 |
| rs9364552 | C | G | 0.035 | 1.50E-05 |
| rs6956990 | C | T | 0.115 | 9.11E-06 |
| rs11509880 | A | G | 0.036 | 2.11E-05 |
| rs2107595^#^ | A | G | 0.074 | 3.41E-13 |
| rs55889159 | A | C | 0.035 | 5.11E-05 |
| rs78850423 | A | G | 0.143 | 9.49E-06 |
| rs2971672 | C | A | 0.036 | 1.90E-05 |
| rs1088868 | G | A | 0.04 | 3.84E-05 |
| rs35146811 | C | A | 0.042 | 5.36E-06 |
| rs112370447 | T | C | 0.045 | 9.62E-07 |
| rs10953541 | C | T | 0.027 | 5.77E-03 |
| rs2024233 | G | A | 0.037 | 2.85E-05 |
| rs11556924^#^ | C | T | 0.067 | 6.26E-13 |
| rs2286198 | G | A | 0.051 | 3.42E-07 |
| rs3918226^#^ | T | C | 0.125 | 1.58E-12 |
| rs2083636 | T | G | 0.051 | 6.44E-08 |
| rs28597716 | A | G | 0.051 | 6.85E-06 |
| rs16885577 | G | A | 0.049 | 2.56E-05 |
| rs10109493 | A | G | 0.061 | 4.93E-05 |
| rs72658939 | G | C | 0.046 | 2.80E-05 |
| rs77211063 | T | C | 0.11 | 2.30E-05 |
| rs10955380 | C | A | 0.04 | 1.19E-05 |
| rs2954029^#^ | A | T | 0.06 | 5.24E-13 |
| rs117938894 | G | A | 0.161 | 3.73E-05 |
| rs75824083 | C | T | 0.907 | 5.09E-05 |
| rs58594043 | A | G | 0.051 | 3.14E-05 |
| rs34914400 | T | C | 0.813 | 1.60E-05 |
| rs2891168^#^ | G | A | 0.173 | 5.23E-104 |
| rs3217992 | T | C | 0.122 | 5.50E-49 |
| rs1333050 | T | C | 0.124 | 2.39E-41 |
| rs4149311 | T | C | 0.052 | 9.06E-06 |
| rs1967604 | A | G | 0.037 | 5.01E-05 |
| rs111245230 | C | T | 0.109 | 8.29E-07 |
| rs781622 | T | C | 0.036 | 2.13E-05 |
| rs77275410 | C | T | 0.061 | 2.55E-05 |
| rs10818583 | A | G | 0.043 | 4.85E-06 |
| rs507666^#^ | A | G | 0.074 | 1.34E-12 |
| rs11257613 | G | A | 0.035 | 1.82E-05 |
| rs7094201 | G | A | 0.062 | 5.77E-05 |
| rs1887318^#^ | T | C | 0.058 | 4.12E-12 |
| rs1870634^#^ | G | T | 0.062 | 5.51E-13 |
| rs1657345 | A | G | 0.081 | 4.58E-12 |
| rs17726488 | T | C | 0.103 | 1.01E-05 |
| rs4691 | T | C | 0.041 | 8.67E-06 |
| rs7098414 | A | C | 0.046 | 2.66E-06 |
| rs2246942^#^ | G | A | 0.076 | 3.51E-16 |
| rs59898454 | A | G | 0.147 | 2.09E-06 |
| rs11191416^#^ | T | G | 0.073 | 5.58E-09 |
| rs12252333 | G | A | 0.047 | 4.90E-06 |
| rs2257129 | C | T | 0.096 | 1.64E-06 |
| rs2281674 | C | G | 0.064 | 4.68E-05 |
| rs28596486 | C | T | 0.052 | 2.91E-06 |
| rs56210063 | C | G | 0.068 | 4.00E-05 |
| rs10840293^#^ | A | G | 0.049 | 6.88E-09 |
| rs11042937 | T | G | 0.011 | 2.02E-01 |
| rs3993105 | T | C | 0.047 | 1.06E-07 |
| rs11462682 | G | A | 0.044 | 8.35E-05 |
| rs146039567 | C | A | 0.149 | 2.20E-05 |
| rs2306029 | T | C | 0.039 | 1.57E-05 |
| rs2727020 | C | G | 0.042 | 4.70E-06 |
| rs12146487 | G | A | 0.048 | 2.28E-05 |
| rs12801636 | G | A | 0.043 | 7.75E-06 |
| rs571353 | C | T | 0.043 | 3.47E-06 |
| rs634552 | G | T | 0.05 | 4.71E-05 |
| rs3133293 | G | T | 0.041 | 3.34E-06 |
| rs17712139 | G | A | 0.041 | 3.62E-05 |
| rs2212437 | A | G | 0.04 | 7.63E-06 |
| rs2839812^#^ | T | A | 0.06 | 1.99E-11 |
| rs567040 | C | T | 0.037 | 6.21E-05 |
| rs964184 | G | C | 0.051 | 4.68E-06 |
| rs3782774 | G | A | 0.036 | 2.17E-05 |
| rs3861086 | C | T | 0.045 | 6.34E-07 |
| rs11170820 | G | C | 0.089 | 2.38E-07 |
| rs56245751 | T | C | 0.061 | 1.13E-05 |
| rs11172113 | C | T | 0.036 | 2.44E-05 |
| rs2229357 | G | A | 0.047 | 3.39E-06 |
| rs6538176 | T | C | 0.047 | 6.24E-06 |
| rs11115214 | C | T | 0.042 | 3.71E-05 |
| rs2681472^#^ | G | A | 0.066 | 7.63E-11 |
| rs10774625^#^ | A | G | 0.064 | 9.22E-14 |
| rs2244608^#^ | G | A | 0.051 | 2.32E-09 |
| rs11057401 | T | A | 0.044 | 1.32E-06 |
| rs11057830 | A | G | 0.069 | 4.24E-09 |
| rs1924981 | T | C | 0.046 | 1.86E-07 |
| rs9591012 | G | A | 0.038 | 1.87E-05 |
| rs73468973 | A | G | 0.043 | 5.68E-05 |
| rs75535189 | C | T | 0.262 | 1.01E-05 |
| rs9515203^#^ | T | C | 0.062 | 6.48E-10 |
| rs4773141 | G | C | 0.059 | 9.46E-10 |
| rs9588107 | A | G | 0.033 | 7.80E-05 |
| rs12867664 | A | G | 0.086 | 1.57E-05 |
| rs17102313 | T | C | 0.615 | 4.64E-05 |
| rs12891473 | C | T | 0.035 | 2.33E-05 |
| rs4506804 | T | G | 0.034 | 3.14E-05 |
| rs3832966 | C | T | 0.037 | 4.67E-06 |
| rs112635299 | G | T | 0.163 | 1.65E-05 |
| rs10139550^#^ | G | C | 0.051 | 1.84E-09 |
| rs113025579 | C | T | 0.125 | 4.94E-05 |
| rs147580454 | C | T | 0.033 | 9.72E-05 |
| rs6494488 | A | G | 0.034 | 1.80E-03 |
| rs72743461^#^ | C | A | 0.071 | 4.81E-12 |
| rs7164479^#^ | T | C | 0.072 | 6.38E-18 |
| rs2083460 | T | C | 0.072 | 1.41E-07 |
| rs2071382^#^ | T | C | 0.062 | 7.14E-13 |
| rs17581137 | A | C | 0.042 | 1.38E-05 |
| rs116082507 | T | C | 1.204 | 4.13E-06 |
| rs7185993 | T | C | 0.036 | 1.23E-05 |
| rs247616 | C | T | 0.044 | 1.01E-06 |
| rs35259348 | C | G | 0.051 | 1.15E-07 |
| rs1050362 | A | C | 0.029 | 4.84E-04 |
| rs9929108 | T | G | 0.047 | 2.32E-07 |
| rs3851738 | C | G | 0.041 | 6.67E-07 |
| rs7500448^#^ | A | G | 0.059 | 5.14E-09 |
| rs1968266 | T | C | 0.037 | 5.23E-05 |
| rs117592425 | A | C | 0.203 | 4.14E-06 |
| rs113348108 | G | A | 0.044 | 2.02E-07 |
| rs8068571 | T | C | 0.042 | 1.76E-05 |
| rs9897596 | T | C | 0.039 | 3.13E-06 |
| rs13723 | G | A | 0.035 | 2.39E-05 |
| rs148720362 | C | T | 0.035 | 5.06E-04 |
| rs1122326 | C | A | 0.05 | 2.76E-06 |
| rs8068844 | C | T | 0.043 | 3.93E-07 |
| rs17608766 | C | T | 0.044 | 6.07E-04 |
| rs46522 | T | C | 0.033 | 9.31E-05 |
| rs4643373 | T | C | 0.046 | 1.20E-06 |
| rs62076439 | T | G | 0.044 | 7.83E-07 |
| rs8068952^#^ | G | C | 0.07 | 1.41E-09 |
| rs7212798 | C | T | 0.063 | 4.37E-08 |
| rs6504218 | G | A | 0.041 | 9.41E-07 |
| rs11077501 | C | T | 0.037 | 2.19E-05 |
| rs75589791 | G | A | 0.065 | 4.58E-05 |
| rs35489971 | A | G | 0.054 | 1.06E-06 |
| rs11654510 | C | A | 0.058 | 9.46E-06 |
| rs7211674 | C | A | 0.034 | 6.14E-05 |
| rs9951447 | C | T | 0.038 | 4.37E-06 |
| rs178002 | G | A | 0.042 | 1.91E-06 |
| rs12922 | A | C | 0.05 | 2.92E-05 |
| rs833509 | C | T | 0.039 | 2.58E-05 |
| rs948937 | A | T | 0.034 | 6.24E-05 |
| rs35614134 | A | C | 0.039 | 2.30E-05 |
| rs663129 | A | G | 0.04 | 1.82E-05 |
| rs116843064 | G | A | 0.159 | 2.87E-07 |
| rs111397563 | T | C | 0.052 | 1.28E-08 |
| rs6511720^#^ | G | T | 0.128 | 7.88E-22 |
| rs2738448 | G | C | 0.034 | 5.04E-05 |
| rs167479 | G | T | 0.04 | 2.26E-06 |
| rs73015715 | T | C | 0.049 | 2.32E-06 |
| rs78030362 | G | A | 0.069 | 5.46E-05 |
| rs10423964 | T | C | 0.039 | 2.38E-05 |
| rs10417115 | C | T | 0.068 | 2.25E-05 |
| rs34322801 | C | G | 0.05 | 6.03E-06 |
| rs73045269 | T | C | 0.064 | 1.71E-07 |
| rs4760 | G | A | 0.054 | 2.56E-05 |
| rs7412^#^ | C | T | 0.143 | 2.17E-19 |
| rs56131196 | A | G | 0.082 | 2.71E-12 |
| rs1964272 | G | A | 0.044 | 2.29E-07 |
| rs425105 | C | T | 0.047 | 4.72E-05 |
| rs13734 | A | G | 0.043 | 2.03E-05 |
| rs59909520 | C | T | 0.059 | 1.04E-05 |
| rs867186 | A | G | 0.057 | 1.47E-05 |
| rs117113213 | A | G | 0.131 | 1.19E-06 |
| rs6129767 | G | T | 0.04 | 1.04E-05 |
| rs56313611 | C | T | 0.058 | 1.39E-06 |
| rs259983 | C | A | 0.056 | 2.89E-06 |
| rs3813452 | T | C | 0.035 | 3.67E-05 |
| rs2832275 | T | A | 0.051 | 2.04E-06 |
| rs75187018 | G | A | 0.136 | 1.85E-05 |
| rs28451064^#^ | A | G | 0.133 | 2.62E-23 |
| rs743339 | C | T | 0.075 | 3.05E-14 |
| rs117696200 | T | G | 0.079 | 1.82E-05 |
| rs2836621 | T | C | 0.033 | 4.68E-05 |
| rs35219138 | C | A | 0.034 | 4.83E-05 |
| rs9604969 | A | G | 0.06 | 5.69E-05 |
| rs71313931 | G | C | 0.039 | 1.87E-05 |
| rs11287675 | C | T | 0.035 | 2.78E-05 |
| rs12485143 | C | T | 0.067 | 2.72E-05 |
| rs468224 | A | G | 0.043 | 8.85E-06 |

Note: “#” indicates 46 SNPs which were genome-wide significant at a p-value of 5×10^-8^ and in low linkage disequilibrium defined according to r^2^<0.001.

Supplemental Table 2. Characteristics of participants

|  | Overall  (n= 77,500) | Low genetic risk  (n= 25,855) | Intermediate genetic risk  (n= 25,828) | High genetic risk  (n= 25,817) |
| --- | --- | --- | --- | --- |
| Age at the start of accelerometry sub-study, years | 56.2 (7.7) | 56.3 (7.9) | 56.3 (7.7) | 56.1 (7.7) |
| Sex, n (%) |  |  |  |  |
| Men | 33,462 (43.2) | 11,305 (43.7) | 11,125 (43.1) | 11,032 (42.7) |
| Women | 44,038 (56.8) | 14,550 (56.3) | 14,703 (56.9) | 14,785 (57.3) |
| Body Mass Index | 26.6 (4.5) | 26.6 (4.5) | 26.6 (4.5) | 26.7 (4.5) |
| Townsend Deprivation Index | -1.9 (2.7) | -1.9 (2.7) | -1.9 (2.7) | -1.9 (2.7) |
| Smoking status, n (%) |  |  |  |  |
| Never | 44,988 (58.0) | 15,019 (58.1) | 14,850 (57.5) | 15,119 (58.6) |
| Previous | 27,491 (35.5) | 9,168 (35.5) | 9,317 (36.1) | 9,006 (34.9) |
| Current | 5,021 (6.5) | 1,668 (6.4) | 1,661 (6.4) | 1,692 (6.5) |
| ST (minutes/day, arithmetic mean) | 640.9 (98.7) | 640.6 (99.2) | 639.9 (98.3) | 642.2 (98.7) |
| Light PA (minutes/day, arithmetic mean) | 294.2 (62.8) | 294.2 (63.1) | 294.7 (62.7) | 293.6 (62.5) |
| MVPA (minutes/day, arithmetic mean) | 73.7 (36.7) | 73.8 (37.0) | 73.7 (36.5) | 73.5 (36.6) |
| ST (minutes/day, geometric mean) | 633.0 (632.3, 633.7) | 632.5 (631.3, 633.8) | 632.1 (630.9, 633.3) | 634.4 (633.1, 635.6) |
| Light PA (minutes/day, geometric mean) | 287.2 (286.7, 287.6) | 287.1 (286.3, 287.9) | 287.7 (286.9, 288.5) | 286.6 (285.8, 287.4) |
| MVPA (minutes/day, geometric mean) | 64.2 (63.9, 64.4) | 64.1 (63.6, 64.6) | 64.3 (63.8, 64.8) | 64.0 (63.6, 64.5) |
| Fruit and vegetable intake (total serving of fresh/dried fruit intake and raw/cooked vegetable intake per week) | 7.2 (3.7) | 7.2 (3.6) | 7.2 (3.6) | 7.2 (3.7) |
| Fish intake (score ranging from 0-6 based on oil fish intake and no oil fish intake) | 3.4 (1.4) | 3.4 (1.4) | 3.4 (1.4) | 3.4 (1.4) |
| Alcohol drinking status |  |  |  |  |
| Never | 1,848 (2.4) | 613 (2.4) | 611 (2.4) | 624 (2.4) |
| Previous | 2,010 (2.6) | 694 (2.7) | 634 (2.5) | 682 (2.6) |
| Current | 34,830 (44.9) | 11,564 (44.7) | 11,604 (44.9) | 11,662 (45.0) |
| Current (three or more times per week) | 38,812 (50.1) | 12,984 (50.2) | 12,979 (50.2) | 12,849 (50.0) |
| Processed and red meat intake, days/week | 0.9 (0.5) | 0.9 (0.5) | 0.9 (0.5) | 0.9 (0.5) |
| Combined diet score | 2.0 (0.9) | 2.0 (0.9) | 2.0 (0.9) | 2.0 (0.9) |
| Use of antihypertensive medication, n (%) | 12,198 (15.7) | 3,762 (14.6) | 4,004 (15.6) | 4,432 (17.2) |
| Use of blood-glucose lowering medication, n (%) | 456 (0.6) | 154 (0.6) | 155 (0.6) | 147 (0.6) |
| Use of cholesterol-lowering medication, n (%) | 9,549 (12.3) | 2,660 (10.3) | 3,140 (12.2) | 3,749 (14.5) |
| Sleep |  |  |  |  |
| ≤5 hours/day | 2,758(3.6) | 885 (3.4) | 919 (3.6) | 954 (3.7) |
| 6 hours/day | 13,662 (17.6) | 4,517 (17.5) | 4,483 (17.4) | 4,662 (18.1) |
| 7 hours/day | 33,307 (43.0) | 11,175 (43.2) | 11,078 (42.9) | 11,054 (42.7) |
| 8 hours/day | 22,902 (29.5) | 7,667 (29.7) | 7,731 (29.8) | 7,504 (29.1) |
| ≥9 hours/day | 4,871 (6.3) | 1,611 (6.2) | 1,617 (6.3) | 1,643 (6.4) |
| Incident cases of coronary heart disease | 1,143 | 277 | 370 | 496 |

Note: Values are means (standard deviations) or number of participants (percentages), unless otherwise indicated.

Supplemental Table 3. Characteristics of participants in the original UK Biobank sample and different sub-samples.

|  | Original UK Biobank sample  (n= 502,411) | Sub-sample 1  (n=20,343) | Analytic sample of  the present study  (n= 75,545) | Sub-sample 2  (n= 56,014) |
| --- | --- | --- | --- | --- |
| Age, years | 56.5 (8.1) | 61.5 (7.4) | 56.2 (7.7) | 64.6 (7.8) |
| Sex, n (%) |  |  |  |  |
| Men | 229,081 (45.6) | 9,937 (48.8) | 32,618 (43.2) | 27,071 (48.3) |
| Women | 273,320 (54.4) | 10,406 (51.2) | 42,927 (56.8) | 28,943 (51.7) |
| Body Mass Index | 27.4 (4.8) | 27.0 (4.6) | 26.6 (4.5) | 26.6 (4.5) |
| Townsend Deprivation Index | -1.3 (3.1) | -2.0 (2.7) | -1.9 (2.7) | -1.9 (2.7) |
| Smoking status, n (%) |  |  |  |  |
| Never | 273,467 (54.8) | 12,159 (60.0) | 43,860 (58.1) | 34,467 (62.2) |
| Previous | 173,022 (34.6) | 7,201 (35.5) | 26,774 (35.4) | 19,009 (34.3) |
| Current | 52,962 (10.6) | 923 (4.5) | 4,928 (6.5) | 1,904 (3.5) |
| Fruit and vegetable intake (total serving of fresh/dried fruit intake and raw/cooked vegetable intake per week) | 7.3 (4.2) | 7.0 (3.4) | 7.2 (3.7) | 7.5 (4.1) |
| Fish intake (score ranging from 0-6 based on oil fish intake and no oil fish intake) | 3.4 (1.4) | 3.5 (1.4) | 3.4 (1.4) | 3.4 (1.4) |
| Alcohol drinking status |  |  |  |  |
| Never | 22,380 (4.5) | 710 (3.5) | 1,806 (2.4) | 1,856 (3.3) |
| Previous | 18,094 (3.6) | 653 (3.2) | 1,976 (2.6) | 1,947 (3.5) |
| Current | 243,101 (48.5) | 9,757 (48.0) | 33,942 (44.9) | 26,805 (48.3) |
| Current (three or more times per week) | 217,172 (53.4) | 9,210 (45.3) | 37,821 (50.1) | 24,949 (44.9) |
| Processed and red meat intake, days/week | 0.9 (0.6) | 0.9 (0.6) | 0.9 (0.5) | 0.8 (0.5) |
| Use of antihypertensive medication, n (%) | 103,985 (20.9) | 4,953 (24.4) | 11,887 (15.7) | 13,833 (24.9) |
| Use of blood-glucose lowering medication, n (%) | 5,611 (1.1) | 180 (0.9) | 447 (0.6) | 441 (0.8) |
| Use of cholesterol-lowering medication, n (%) | 86,878 (17.5) | 4,841 (23.8) | 9,282 (12.3) | 13,888 (25.0) |
| Sleep |  |  |  |  |
| ≤5 hours/day | 27,587 (5.5) | 897 (4.5) | 2,701(3.6) | 2,946 (5.4) |
| 6 hours/day | 95,637 (19.2) | 3,542 (17.4) | 13,326 (17.6) | 10,691 (19.2) |
| 7 hours/day | 192,346 (38.6) | 7,939 (39.0) | 32,468 (43.0) | 21,914 (39.4) |
| 8 hours/day | 144,274 (29.0) | 6,222 (30.6) | 22,309 (29.5) | 15,853 (28.5) |
| ≥9 hours/day | 38,341 (7.7) | 1,734 (8.5) | 4,741 (6.3) | 4,177 (7.5) |

Note: Values are means (standard deviations) or number of participants (percentages), unless otherwise indicated. ‘Original UK Biobank sample’ included participants who provided data at baseline (2006-2010); ‘Sub-sample 1’ included participants who had both baseline (2006-2010) and the first repeat-assessment (2012-2013); ‘Analytic sample of the present study’ included participants who had both baseline (2006-2010) and accelerometry data (2013-2015); and ‘Sub-sample 2’ included participants who had both baseline (2006-2010) and the second repeat-assessment (2014+).

Supplemental Methods 1. A Compositional Data Analysis for physical activity, sedentary time and sleep

**Isometric log-ratio transformation**

Log-ratio transformation of compositional variables ensures the relationships between different compositions to be correctly defined. Amongst many different types of log-transformed ratios used in compositional data analysis methods, isometric log-ratio (ILR) pivot coordinates are widely adopted in explaining movement behavior. It accounts for the compositional nature of the data by producing orthonormal basis of compositional data, thereby enabling isometric mapping (i.e., the relative distance between compositional data points is preserved to the real space). The ILR pivot variables used in this study are calculated as follows. Since we have 4 components included in the original composition, 3 ILR-transformed variables are obtained:

ILR-Coordinate_1_ =√3/4 ln(MVPA/(ST×LPA×Sleep)^1/3^)

ILR-Coordinate_2_ = √2/3 ln(ST / (LPA×Sleep)^1/2^)

ILR-Coordinate_3_ = √1/2 ln(LPA / Sleep)

The three ILR pivot coordinates are included in survival models in replacement to the raw movement time variables. The coefficients in the fitted model are more complicated to interpret owing to their relation to isometric log-ratio pivot coordinates rather than raw behavior variables. The first coordinate indicates the balance between MVPA and all the other behaviors, meaning the effect of reallocating time spent in the sum of LPA, ST and Sleep to MVPA. The second and third may not be straightforward to interpret. Therefore, we ran three models, with each model having each behavior as the numerator in calculating the first ILR-coordinate (LPA, MVPA and ST – models using sleep as the pivot coordinate were not performed due to its known non-linear, U-shaped association with cardiovascular disease). Then the coefficients of the first ILR pivot coordinates are reported in Table 1.

Using the same set of ILR coordinates, isotemporal substitutional analyses were also carried out as mentioned in the main text, Figure 2 and Figure 3. Any zero values in movement time (11 observations from time in moderate-to-vigorous physical activity; less than 0.01%) were imputed with a small positive value (0.00000001) to avoid undefined value in the calculation of the coordinates.


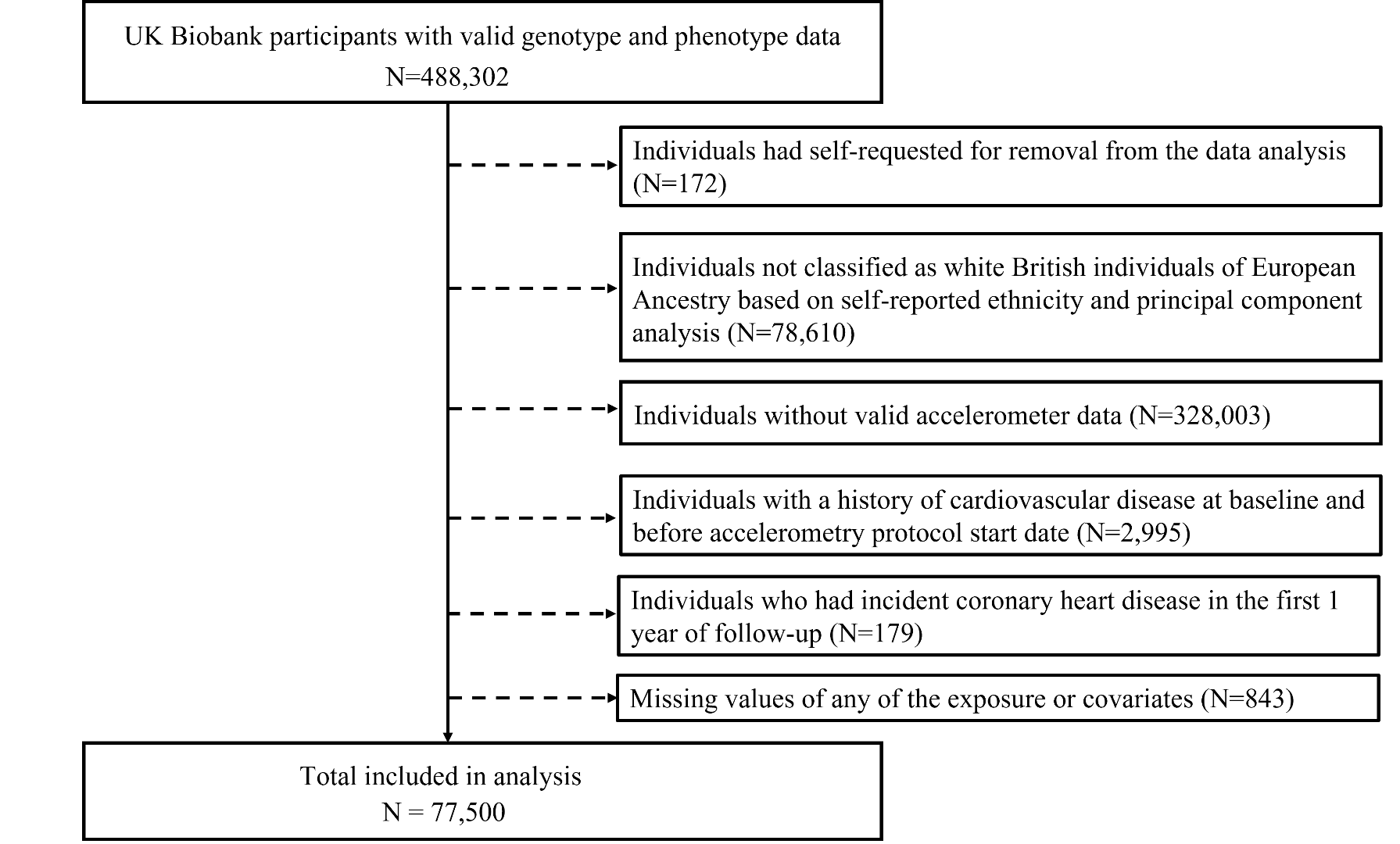


Supplemental Figure 1. Participant flow chart.


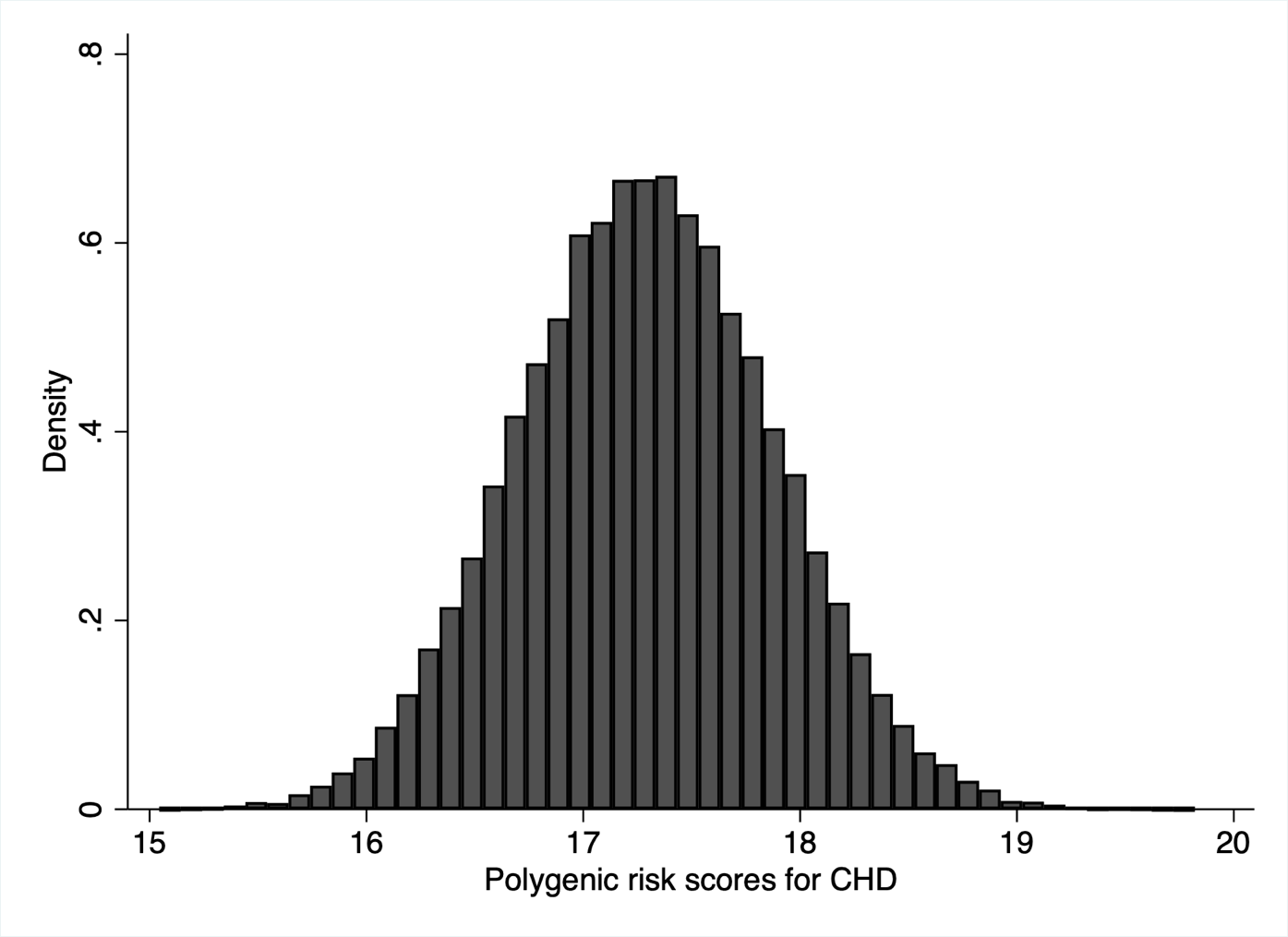


Supplemental Figure 2. Distribution of individuals by calculated weighted polygenic risk scores for coronary heart disease (CHD).


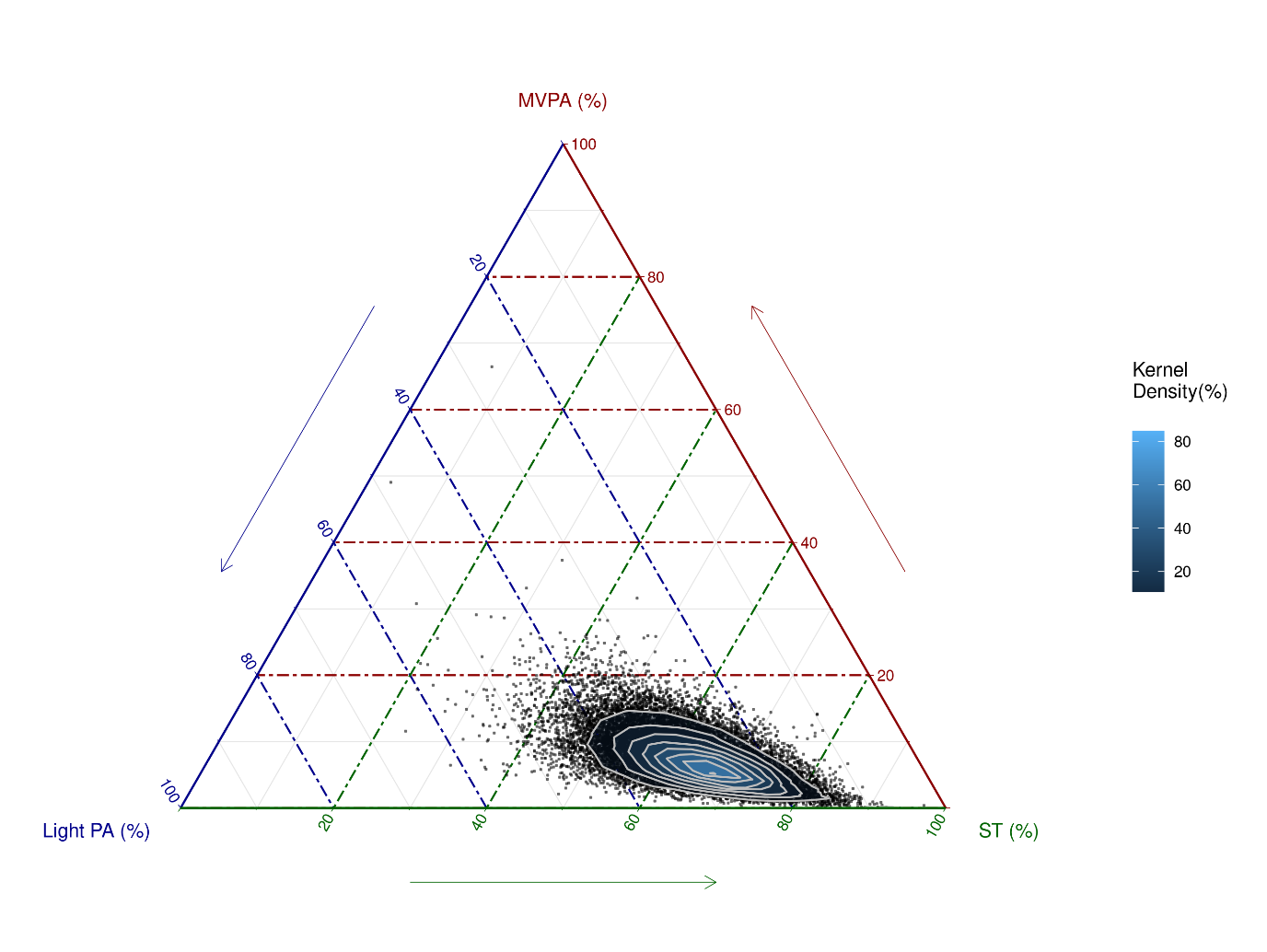


Supplemental Figure 3. A compositional ternary heat map plot indicating the distribution of individuals according to relative proportions of time spent on sedentary time, light physical activity, and moderate-to-vigorous physical activity.

Abbreviations: ST = sedentary time; Light PA = light physical activity; and MVPA = moderate-to-vigorous physical activity


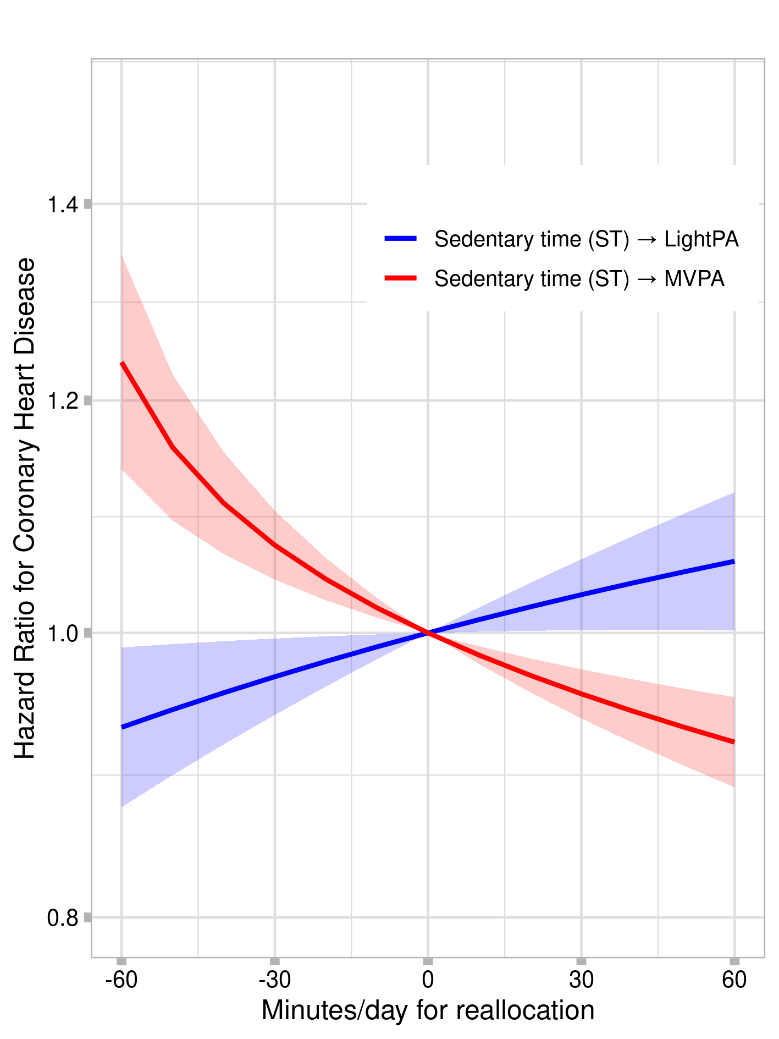


Supplemental Figure 4. Hazard ratios of coronary heart disease estimated from reallocating sedentary time into physical activity time (and vice versa), while keeping the remaining components constant, after excluding an additional 1 year of follow-up to address potential for reverse causality. Notes: Cox regression models using compositional isotemporal substitution modelling with age (at the start of the accelerometry sub-study) as the underlying timescale (not as a confounder) were adjusted for sex, smoking status (never, previous, current), alcohol consumption (never, previous, currently < 3 times/week, currently ≥ 3 times/week), diet (a combined score based on the number of increased intake of vegetable, fruit, and fish and decreased intake of processed and red meat, with a greater score indicating a more favorable dietary habit), Townsend Deprivation Index (a composite score of employment, car ownership, home ownership and household overcrowding; based on postcode, with higher values indicating a higher degree of deprivation), use of antihypertensive medication and use of blood-glucose lowering medication, genotype array type and first ten principal components of genetic ancestry, with mutual adjustment for the three ILR terms (i.e. three ILRs of sedentary time included in models for sedentary time; three ILRs of light PA included in models for light PA; and three ILRs of MVPA included in models for MVPA).

Abbreviations: ST = sedentary time; Light PA = light physical activity; and MVPA = moderate-to-vigorous physical activity.


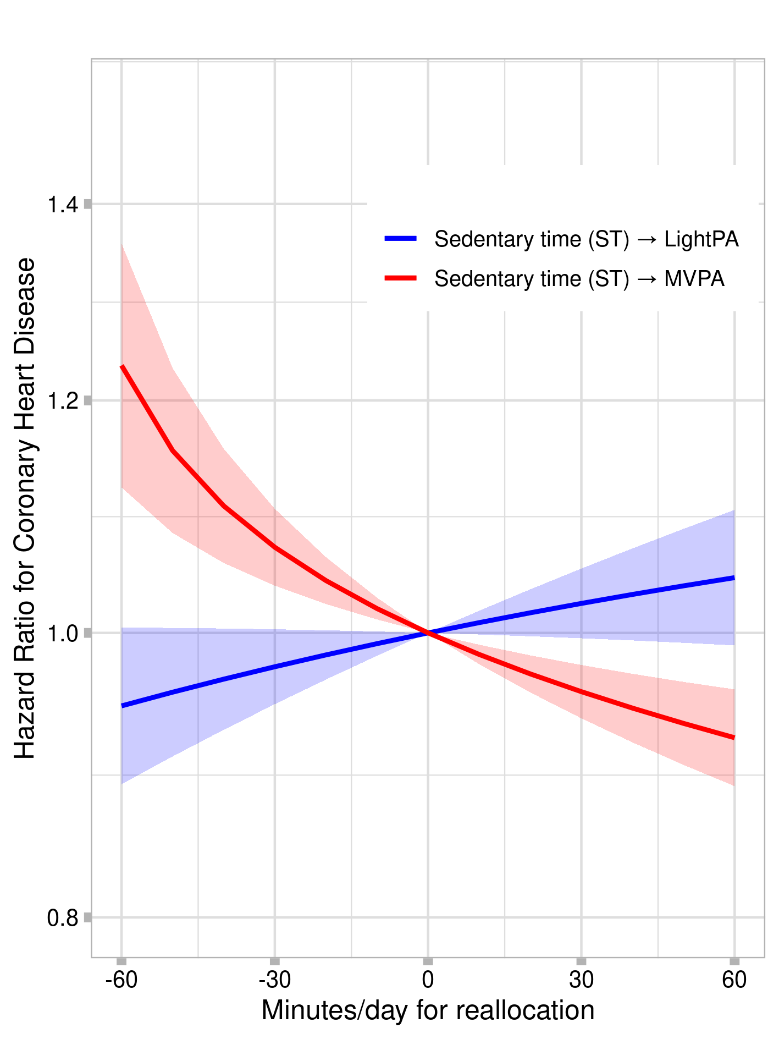


Supplemental Figure 5. Hazard ratios of coronary heart disease estimated from reallocating sedentary time into physical activity time and physical activity time into sedentary time, while keeping the remaining components constant after excluding individuals with the 2^nd^-degree genetic relatedness, Notes: Cox regression models using compositional isotemporal substitution modelling with age (at the start of the accelerometry sub-study) as the underlying timescale (not as a confounder) were adjusted for sex, smoking status (never, previous, current), alcohol consumption (never, previous, currently < 3 times/week, currently ≥ 3 times/week), diet (a combined score based on the number of increased intake of vegetable, fruit, and fish and decreased intake of processed and red meat, with a greater score indicating a more favorable dietary habit), Townsend Deprivation Index (a composite score of employment, car ownership, home ownership and household overcrowding; based on postcode, with higher values indicating a higher degree of deprivation), use of antihypertensive medication and use of blood-glucose lowering medication, genotype array type and first ten principal components of genetic ancestry, with mutual adjustment for the three ILR terms (i.e. three ILRs of sedentary time included in models for sedentary time; three ILRs of light PA included in models for light PA; and three ILRs of MVPA included in models for MVPA).

Abbreviations: ST = sedentary time; Light PA = light physical activity; and MVPA = moderate-to-vigorous physical activity.


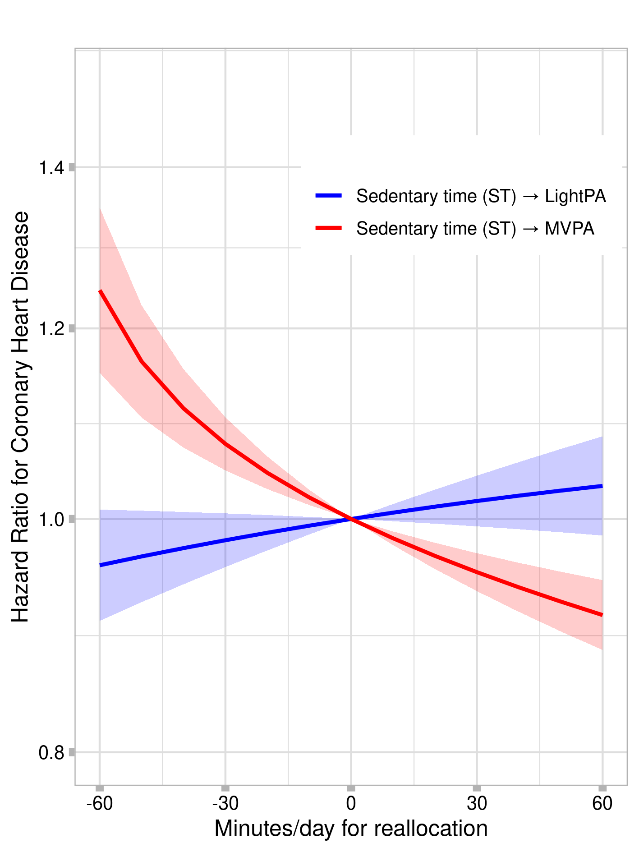

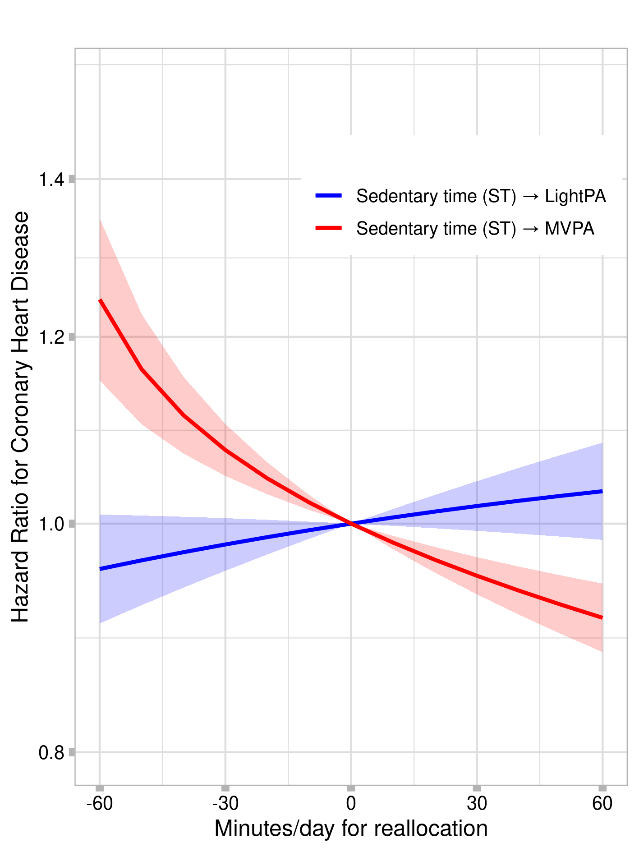


Supplemental Figure 6. Hazard ratios of coronary heart disease estimated from reallocating sedentary time into physical activity time and physical activity time into sedentary time, while keeping the remaining components constant, using a different set of ENMO cut-offs to define the movement behaviors (Left panel: milli-g ≤25 for ST and milli-g ≥125 for MVPA; Right panel: milli-g ≤35 for ST and milli-g ≥125 for MVPA). Notes: Cox regression models using compositional isotemporal substitution modelling with age (at the start of the accelerometry sub-study) as the underlying timescale (not as a confounder) were adjusted for sex, smoking status (never, previous, current), alcohol consumption (never, previous, currently < 3 times/week, currently ≥ 3 times/week), diet (a combined score based on the number of increased intake of vegetable, fruit, and fish and decreased intake of processed and red meat, with a greater score indicating a more favorable dietary habit), Townsend Deprivation Index (a composite score of employment, car ownership, home ownership and household overcrowding; based on postcode, with higher values indicating a higher degree of deprivation), use of antihypertensive medication and use of blood-glucose lowering medication, genotype array type and first ten principal components of genetic ancestry, with mutual adjustment for the three ILR terms (i.e. three ILRs of sedentary time included in models for sedentary time; three ILRs of light PA included in models for light PA; and three ILRs of MVPA included in models for MVPA).

Abbreviations: ST = sedentary time; Light PA = light physical activity; and MVPA = moderate-to-vigorous physical activity.


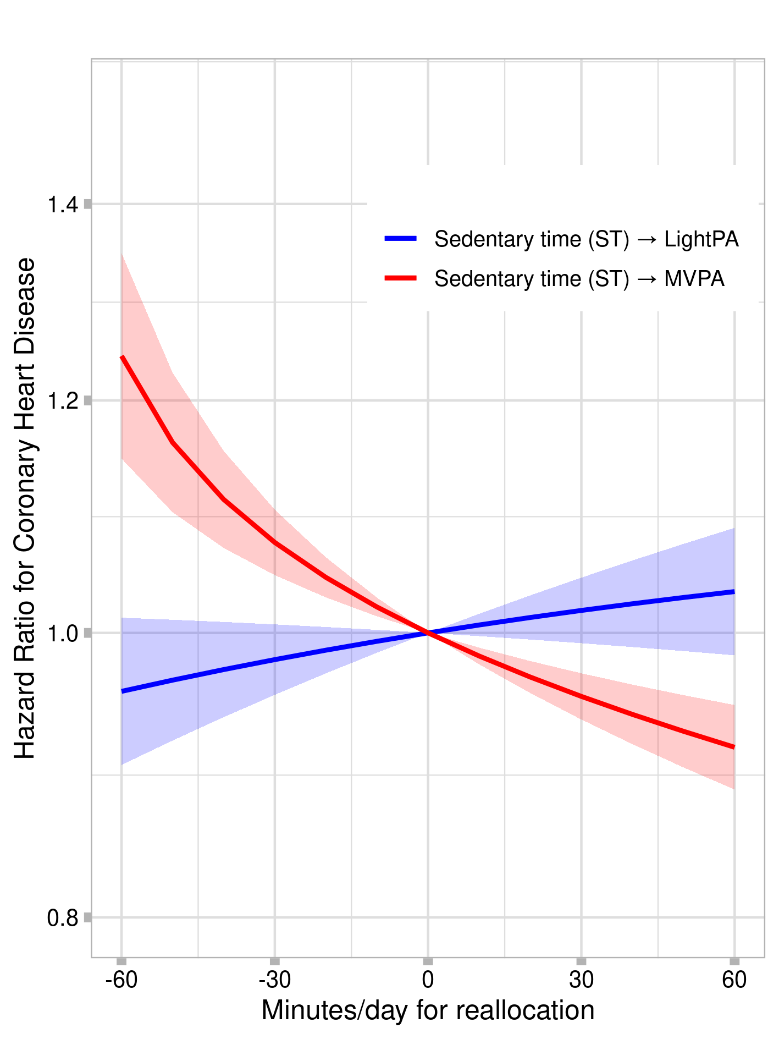


Supplemental Figure 7. Hazard ratios of coronary heart disease estimated from reallocating sedentary time into physical activity time and physical activity time into sedentary time, while keeping the remaining components constant, using missing values imputed using multiple imputation by chained equations. Notes: Cox regression models using compositional isotemporal substitution modelling with age (at the start of the accelerometry sub-study) as the underlying timescale (not as a confounder) were adjusted for sex, smoking status (never, previous, current), alcohol consumption (never, previous, currently < 3 times/week, currently ≥ 3 times/week), diet (a combined score based on the number of increased intake of vegetable, fruit, and fish and decreased intake of processed and red meat, with a greater score indicating a more favorable dietary habit), Townsend Deprivation Index (a composite score of employment, car ownership, home ownership and household overcrowding; based on postcode, with higher values indicating a higher degree of deprivation), use of antihypertensive medication and use of blood-glucose lowering medication, genotype array type and first ten principal components of genetic ancestry, with mutual adjustment for the three ILR terms (i.e. three ILRs of sedentary time included in models for sedentary time; three ILRs of light PA included in models for light PA; and three ILRs of MVPA included in models for MVPA).

Abbreviations: ST = sedentary time; Light PA = light physical activity; and MVPA = moderate-to-vigorous physical activity.


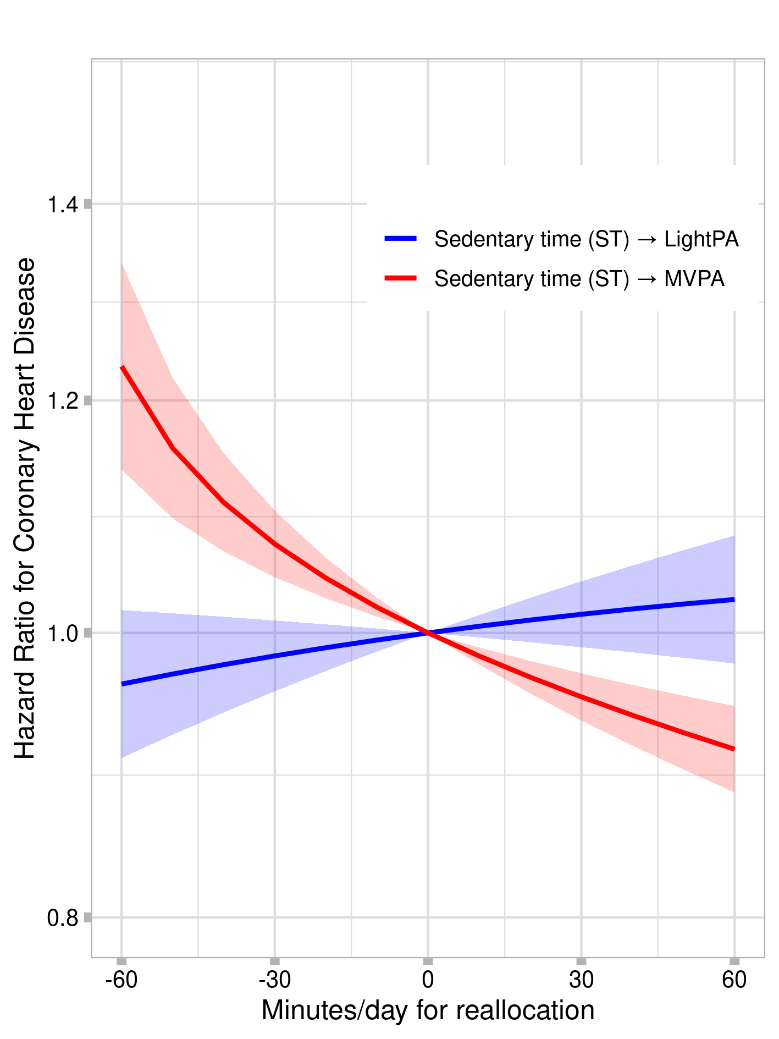


Supplemental Figure 8. Hazard ratios of coronary heart disease estimated from reallocating sedentary time into physical activity time and physical activity time into sedentary time, while keeping the remaining components constant, using a weighted polygenic risk score calculated based only on 46 lead SNPs from 46 loci after applying a more stringent LD cut-off point of 0.001 in order to minimize over-estimation of effect size of SNPs. Notes: Cox regression models using compositional isotemporal substitution modelling with age (at the start of the accelerometry sub-study) as the underlying timescale (not as a confounder) were adjusted for sex, smoking status (never, previous, current), alcohol consumption (never, previous, currently < 3 times/week, currently ≥ 3 times/week), diet (a combined score based on the number of increased intake of vegetable, fruit, and fish and decreased intake of processed and red meat, with a greater score indicating a more favorable dietary habit), Townsend Deprivation Index (a composite score of employment, car ownership, home ownership and household overcrowding; based on postcode, with higher values indicating a higher degree of deprivation), use of antihypertensive medication and use of blood-glucose lowering medication, genotype array type and first ten principal components of genetic ancestry, with mutual adjustment for the three ILR terms (i.e. three ILRs of sedentary time included in models for sedentary time; three ILRs of light PA included in models for light PA; and three ILRs of MVPA included in models for MVPA).

Abbreviations: ST = sedentary time; Light PA = light physical activity; and MVPA = moderate-to-vigorous physical activity.


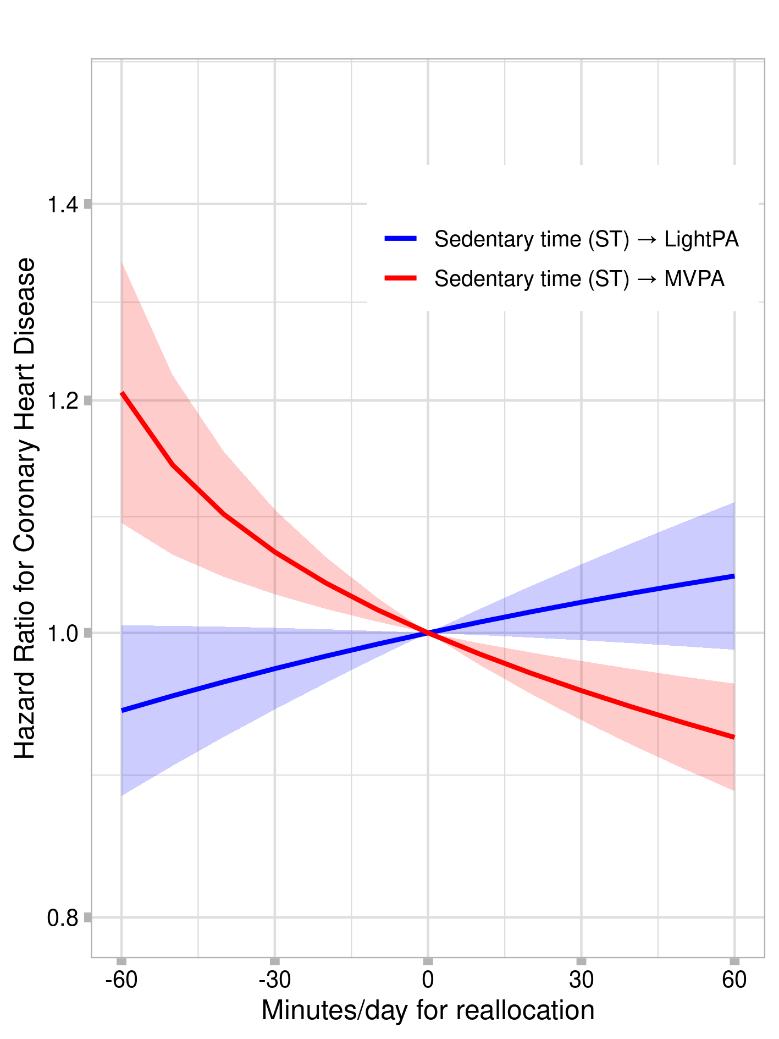


Supplemental Figure 9. Hazard ratios of coronary heart disease estimated from reallocating sedentary time into physical activity time and physical activity time into sedentary time, while keeping the remaining components constant after excluding individuals with prevalent cancer at baseline. Notes: Cox regression models using compositional isotemporal substitution modelling with age (at the start of the accelerometry sub-study) as the underlying timescale (not as a confounder) were adjusted for sex, smoking status (never, previous, current), alcohol consumption (never, previous, currently < 3 times/week, currently ≥ 3 times/week), diet (a combined score based on the number of increased intake of vegetable, fruit, and fish and decreased intake of processed and red meat, with a greater score indicating a more favorable dietary habit), Townsend Deprivation Index (a composite score of employment, car ownership, home ownership and household overcrowding; based on postcode, with higher values indicating a higher degree of deprivation), use of antihypertensive medication and use of blood-glucose lowering medication, genotype array type and first ten principal components of genetic ancestry, with mutual adjustment for the three ILR terms (i.e. three ILRs of sedentary time included in models for sedentary time; three ILRs of light PA included in models for light PA; and three ILRs of MVPA included in models for MVPA).

Abbreviations: ST = sedentary time; Light PA = light physical activity; and MVPA = moderate-to-vigorous physical activity.
